# Imaging-guided treatment strategy compared to standard care in at-risk individuals for rheumatoid arthritis: a cost-utility analysis

**DOI:** 10.1136/bmjopen-2026-118495

**Published:** 2026-10-21

**Authors:** Sterre Bour, Elise Van Mulligen, Lucas Goossens, Pascal Hendrik Pieter de Jong, Annette van der Helm - van Mil, Maureen Rutten-van Molken

**Affiliations:** 1 Erasmus School of Health Policy & Management, Erasmus Universiteit Rotterdam, Rotterdam, The Netherlands; 2 Department of Rheumatology, Leiden University Medical Center, Leiden, The Netherlands; 3 Department of Rheumatology, Erasmus MC University Medical Center Rotterdam, Rotterdam, The Netherlands; 4 Institute for Medical Technology Assessment, Erasmus Universiteit Rotterdam, Rotterdam, The Netherlands

**Keywords:** HEALTH ECONOMICS, RHEUMATOLOGY, Diagnostic Imaging

## Abstract

**Abstract:**

**Objective:**

Some individuals present with symptoms before rheumatoid arthritis (RA) develops, and trials suggest that early treatment during this at-risk phase may be effective. This study evaluated the cost-utility of three imaging strategies—conventional MRI, modified Dixon MRI and ultrasound—combined with early treatment compared with watchful waiting in individuals at risk of RA, stratified by autoantibody status.

**Design:**

A decision tree combined with a Markov model was developed to estimate costs and quality-adjusted life years (QALYs) over a 2-year time horizon. Model inputs were derived from the TREAT EARLIER trial for treated patients at risk of developing RA, clinically suspect arthralgia (CSA) cohorts for untreated at-risk patients and the early arthritis clinic for patients with undifferentiated arthritis or RA. Cost-utility analyses were conducted from both the societal- and healthcare perspectives. Probabilistic and deterministic sensitivity analyses were conducted to assess the uncertainty of the results, and several scenarios were explored.

**Setting:**

Dutch hospital setting.

**Participants:**

Individuals at risk of developing RA, stratified by autoantibody status.

**Interventions:**

Watchful waiting was compared with three imaging strategies—conventional MRI, modified Dixon MRI and ultrasound—combined with early treatment (single intramuscular glucocorticoid injection and 1-year methotrexate course) in patients with positive subclinical inflammation.

**Outcome measure:**

Incremental cost-effectiveness ratios (ICERs) of the interventions compared with watchful waiting. Cost-effectiveness probabilities were estimated at a willingness-to-pay (WTP) threshold of €50,000 per QALY.

**Results:**

From a societal perspective, autoantibody-positive patients showed cost savings of €1,183 (-5348 to 2861) (ultrasound) to €1,825 (-8041 to 3861) (modified Dixon MRI). Autoantibody-negative patients had incremental costs from €21 (-3843 to 3856) (conventional MRI) to -€409 (-4499 to 3681) (modified Dixon MRI). QALYs gained ranged from 0.006 (-0.04 to 0.06) to 0.016 (-0.08 to 0.11). Probability of cost-effectiveness was 75%–80% for autoantibody-positive patients and 56%–70% for autoantibody-negative patients at a WTP of €50 000/QALY. Results were less pronounced from a healthcare perspective. Deterministic sensitivity analysis showed the results were robust to most changes in parameters; however, the results were sensitive to extreme changes in parameters related to treatment effects on quality of life (QoL) and productivity and disutilities due to treatment-related side effects.

**Conclusions:**

Imaging-guided early treatment for arthralgia at-risk for RA may be cost-effective, particularly for autoantibody-positive patients, irrespective of imaging modality. Modified Dixon MRI seems most promising and requires further validation in CSA.

**Trial registration:**

TREAT EARLIER trial: EudraCT, 2014–004472-35 and the Netherlands Trial Register, NTR4853-trial-NL4599.

**STRENGTHS AND LIMITATIONS OF THIS STUDY**

- This study models the full disease pathway from at-risk state through the diagnostic phase to established rheumatoid arthritis (RA), integrating data from pre-RA and early RA cohorts.
- The model was informed by patient-level data from the TREAT EARLIER trial and observational cohorts, allowing subgroup analyses by autoantibody status and improving the precision of transition probabilities, costs and utilities.
- The time horizon was limited to 2 years because at the time of this study, no follow-up data were available on the long-term effects of early treatment after progression to RA.
- In the absence of modality-specific outcome data for modified Dixon MRI and ultrasound, outcomes were extrapolated from conventional MRI, and thus comparisons between imaging modalities should be interpreted with caution.

## Introduction

 Rheumatoid arthritis (RA) is a chronic autoimmune disease characterised by chronic inflammatory arthritis (IA), leading to joint damage, disability and increased morbidity.^1^ It affects approximately 1% of the population in North America and Europe,^2^ with around 17,400 new cases annually in the Netherlands.^3^ After diagnosis, RA is treated with disease-modifying antirheumatic drugs.^4^ Early and intensive treatment improves long-term outcomes,^5^ while inadequate management can cause high healthcare and societal burden.^6 7^ Consequently, treatment within 12 weeks is recommended.^8^

Currently, RA is detected when swollen joints are recognised by a rheumatologist. However, some individuals experience symptoms in an earlier phase, and autoimmunity can already develop years before the development of RA.^9^ Individuals who experience symptoms suspicious for RA, without detected swollen joints, are so-called individuals with clinically suspect arthralgia (CSA).^10^ Approximately 20% of the individuals with CSA develop RA, but it remains challenging to accurately predict who.^11^ Several studies have shown the predictive value of serological biomarker tests, in particular rheumatoid factor (RF) and/or anti-citrullinated protein antibodies (ACPA).^11 12^ Ultrasound and MRI can detect subclinical joint inflammation in the hands and feet, which is also predictive for the development of RA.^13–18^ However, since the added value of imaging relative to its costs is unknown and a positive result has no direct therapeutic consequence, imaging is currently not part of Dutch and international guidelines for diagnosing RA at an earlier stage.

Different proof-of-concept clinical trials showed the effectiveness of treatment in individuals with CSA at higher risk of developing RA (ie, autoantibody-positive and/or a positive subclinical inflammation score) in terms of reduced pain, improved productivity and lower detected subclinical inflammation scores.^19–22^ A trial-based cost-utility analysis (CUA) of the TREAT EARLIER trial showed that treatment of individuals with CSA, who showed subclinical inflammation on the MRI, is likely to be cost-effective.^23^ However, the intervention was not compared with the current standard of care (ie, watchful waiting) but with a strategy in which patients had an MRI without a therapeutic consequence. Since this is not common practice, the value of imaging in the at-risk phase should be determined.

In the Netherlands, the Dutch Society for Rheumatology (NVR) develops clinical guidelines for diagnostics and treatment of RA based on guidelines and recommendations of the European League against Rheumatism (EULAR).^24^ For imaging-based detection and treatment strategies to be included in these guidelines, evidence is needed on both the effectiveness and cost-effectiveness. The aim of this study is to assess the cost-utility of three imaging strategies–conventional MRI, modified Dixon MRI and ultrasound–followed by early treatment of patients with a positive imaging result, compared with watchful waiting, for individuals at risk of developing RA.

## Methods

We developed a health economic model, adhering to the guidelines of the International Society of Pharmacoeconomic and Outcomes Research-Society of Medical Decision Making task force on good research practices for economic modelling^25^ and the Dutch guideline for economic evaluations in healthcare.^26^ The analysis was reported conforming to the Consolidated Health Economic Evaluation Reporting Standards (CHEERS) checklist,^27^ which for transparency is presented in online supplemental table S1.

### Model identification and scope

#### Study population, setting and location

The target population consisted of adults with CSA, without detectable swollen joints, as identified by rheumatologists in the Netherlands.

The study population was stratified into autoantibody-positive (ie, RF and/or ACPA positive) and autoantibody-negative patients. This stratification was applied because these two subgroups have differences in underlying pathophysiology, as well as differences in the diagnostic value of imaging modalities and in treatment effects.^28^ Autoantibody-positive and -negative patients were assumed to have the same demographic characteristics. This study reflected the Dutch healthcare setting.

Individuals at risk of IA (in this case being either undifferentiated arthritis or RA) either developed IA within 2 years or they did not. The mean age of individuals with CSA who did not develop IA was 44 and the male/female ratio was 21%/79%. Those who developed IA had a mean age of 48 and a male/female ratio of 28%/72%.

#### Intervention: diagnostics & treatment

Three imaging strategies–US, conventional MRI and modified Dixon MRI–were evaluated alongside standard diagnostics,^29^ see table 1 for details.

**Table 1:** Description of three imaging modalities

Ultrasound	Ultrasound is a valuable imaging modality for detecting synovitis in patients with RA, due to its widespread availability, relatively low costs and safety; it is routinely used in monitoring RA.^13 17 62^ However, ultrasound is operator dependent, the interpretation of the images is more subjective and the quality of ultrasound images is generally lower compared with other imaging modalities.^63^ To facilitate generalisability of ultrasound findings, a standardised, consensus-based scoring system was developed.^64 65^ The value of ultrasound is shown in various studies, especially for patients demonstrating a positive power doppler ultrasound tenosynovitis signal.^14 15 17^ However, the cost-effectiveness of ultrasound as an additive tool in the diagnostic process of IA is unknown. The mean costs for an ultrasound scan were €103.^34^
Conventional MRI	MRI improves sensitivity and reproducibility in the early detection of synovitis and tenosynovitis compared with US.^13 66 67^ The development of the rheumatoid arthritis MRI scoring system (RAMRIS) by OMERACT improved the interpretability and comparability of MRI findings.^68^ However, MRI is less readily available and has high costs. These costs can be reduced, since a recent study showed that only scanning the hand and wrist joints yields comparable predictive accuracy compared with also scanning the feet.^18^ The scan time of the conventional MRI is 30–40 min. The cost-effectiveness of MRI is unknown. The mean costs for MRI were €267 per scan.^13^ We modelled scanning both hands because for the other imaging modalities also both hands were scanned, thus increasing the costs to €534.
Modified Dixon MRI	The modified Dixon MRI uses an innovative fat suppression technique with a reduced scan time of 6 min compared with the conventional MRI, and it also does not need a contrast agent.^46^ A recent study demonstrated comparable diagnostic performance between modified Dixon MRI and conventional MRI in an early arthritis cohort.^46^ The setting of CSA is characterised by subclinical joint inflammation (thus less severe inflammation than in the RA phase). The accuracy of modified Dixon MRI in the CSA risk phase remains to be validated. The estimated costs by a radiologist for the contrast agent were €100. So, the estimated costs for a modified Dixon scan were based on expert opinion and were estimated at €110 for two hands (based on the reduced scan time and no need for the contrast agent).

CSA, clinically suspect arthralgia; IA, inflammatory arthritis; RA, rheumatoid arthritis; US, ultrasound.

In the intervention strategy, positive imaging led to treatment with a single intramuscular glucocorticoid injection (120 mg) and a 1 year course of oral methotrexate (MTX) (up to 25 mg/week, adjusted for side effects).^21 30^ If individuals developed IA within 1 year, MTX was continued. If individuals did not develop IA within 1 year, treatment was stopped until swollen joints were detected by the rheumatologist.

#### Comparator: current diagnostics and treatment strategy

The intervention strategies were compared with each other and separately to watchful waiting. Watchful waiting was based on the Dutch 2015 guideline to diagnose RA, which remains the most current diagnostic guideline in use.^29^ According to this guideline, first-line treatment with MTX should not be initiated unless swollen joints were detected based on physical examination of the rheumatologist. In this guideline, testing for autoantibodies associated with RA (ACPA and RF) and non-antibody serological markers (C-reactive protein (CRP) and erythrocyte sedimentation rate (ESR))^29^ is recommended, but positive tests do not have a therapeutic consequence.

#### Time horizon, perspective of the analysis and costs

The time horizon was 2 years to focus on the immediate effects of the diagnostic phase, and the cycle length was 4 months. Half-cycle correction was applied to assume that, on average, transitions occur halfway through each cycle. The analysis was conducted from both the societal and healthcare perspectives within the Dutch healthcare setting. The healthcare perspective incorporated direct and indirect medical costs, regardless of the relation to the disease of interest. In the societal perspective, productivity costs and informal care costs were also considered.^31 32^ The direct and indirect medical costs and informal care costs were based on resource use reported in the iMTA medical consumption questionnaire,^33^ and valued in 2022 euros using unit costs reported in the Dutch costing manual.^34^ Productivity costs were derived from the work productivity and activity impairment questionnaire.^35^ The mean hourly wage applied to productivity losses was €39.88.^34^ The annual discount rates (r) used were 3% for costs and 1.5% for effects.

#### Type of economic evaluation and outcome measures

A model-based CUA was conducted, with the primary outcome being the incremental cost-effectiveness ratio (ICER) calculated by dividing the incremental costs by the incremental quality-adjusted life years (QALYs) over 2 years. QALYs were calculated using utilities from the EuroQol-5 dimensions-5 levels (EQ-5D-5L) questionnaire based on the Dutch tariff.^36^ A willingness to pay (WTP) threshold of €50 000/QALY was used.^23^ Other primary outcomes included the net monetary benefit (NMB) and incremental net monetary benefit (iNMB). The NMB was calculated by multiplying QALYs by the WTP threshold of €50 000 per QALY and subtracting the total costs of the strategy. The iNMB was defined as the difference in NMB between competing strategies.

### Model structure

We developed a health economic model that consisted of a decision tree linked to a Markov model to evaluate diagnostic strategies for patients with CSA, stratified by autoantibody status (ACPA or RF positive, or ACPA and RF negative) (figure 1). These biomarkers were measured by default for every patient with CSA, irrespective of the imaging strategy, which included:

1. **Watchful waiting** (ie, usual care): Individuals were monitored without treatment until swollen joints were detected with physical examination by the rheumatologist.
2. **Conventional MRI strategy**: conventional MRI of the hands and wrists, with early treatment initiated for those with positive findings. Patients with a negative MRI did not start early treatment until swollen joints were identified with physical examination by the rheumatologist.
3. **Modified Dixon MRI strategy**: This strategy was the same as the conventional MRI strategy, only different costs were allocated to the modified Dixon MRI and higher uncertainty was modelled for the diagnostic accuracy of the modified Dixon MRI.
4. **Ultrasound strategy:** Ultrasound examination, with early treatment initiated for those with a positive result. Patients with a negative ultrasound did not start treatment until swollen joints were identified by physical examination.

**Figure 1:**
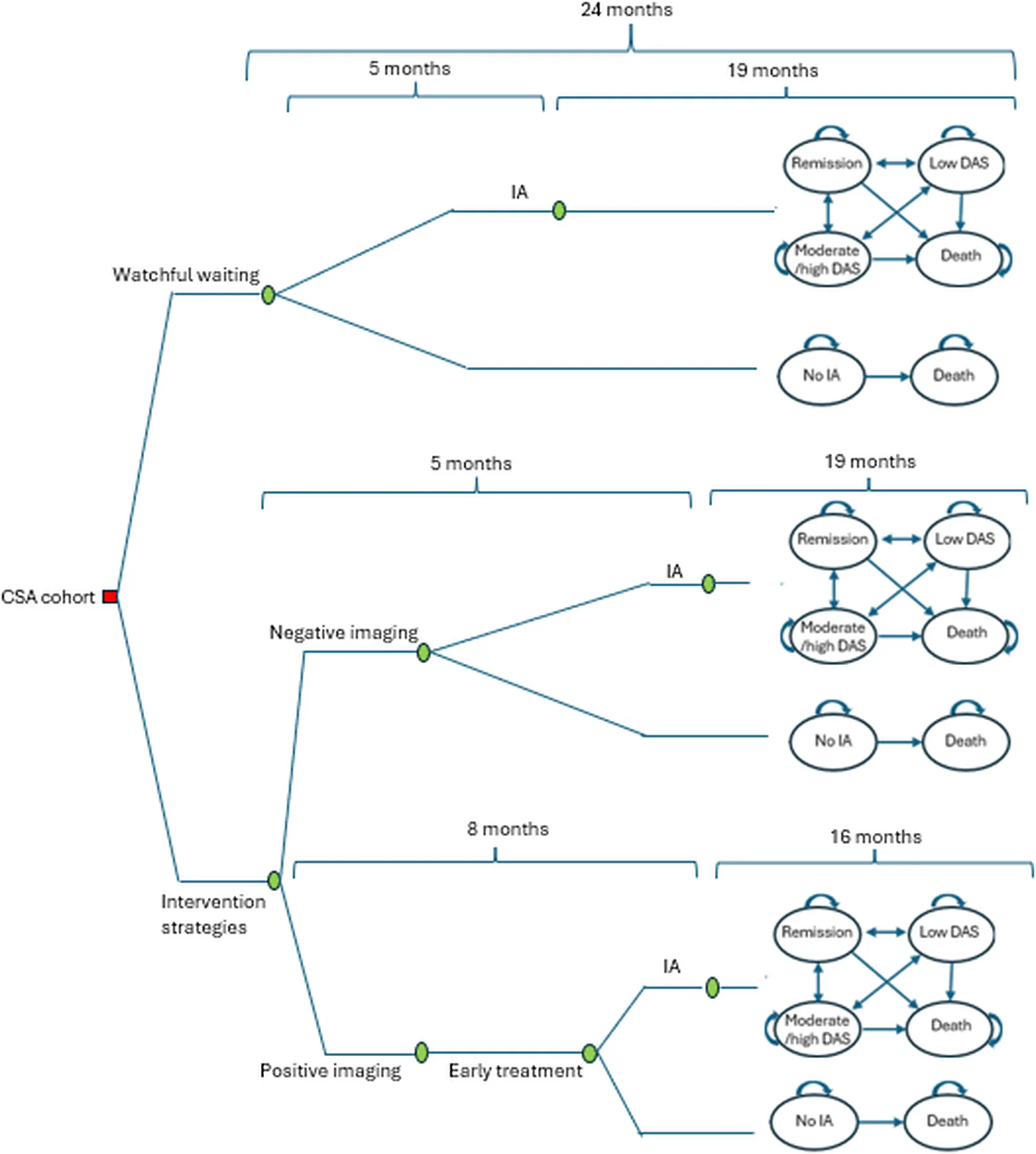
Schematic overview of the model. Note: The model used a total time horizon of 24 months. Patients entered a decision tree followed by a Markov model, with the timing of transition depending on the strategy and whether inflammatory arthritis (IA) developed. In the watchful waiting strategy, patients who developed IA did so after an average of 5 months. These individuals remained in the decision tree for 5 months and then entered the Markov model for the remaining 19 months. On entry, they could transition into one of four health states: remission, low disease activity (DAS), moderate/high DAS or death. Patients who did not develop IA entered the Markov model immediately in the ‘No IA’ health state for 24 months and were only subject to background mortality. In the intervention strategies, patients with a positive imaging result who received early treatment experienced a delay in IA onset of 3 months compared with untreated patients (see paragraph 2.4.1). As a result, IA developed after an average of 8 months, after which patients entered the Markov model for the remaining 16 months. Patients with a negative imaging result followed the same pathway as in the watchful waiting strategy, with an average time to IA development of 5 months if IA occurred or immediate entry into the ‘No IA’ state otherwise. *Red square=decision node, green circle=chance node, triangle=end node, CSA, clinically suspect arthralgia; DAS, disease activity score; IA, inflammatory arthritis; MD, modified Dixon; US, ultrasound.

Patients who developed IA remained in the decision tree until they progressed to IA. 45% of the autoantibody-positive and 9% of the autoantibody-negative patients developed IA. The mean time to IA development was 5 months for patients with untreated CSA, based on CSA cohorts in Leiden^37^ and Rotterdam.^38^

Patients who developed IA entered the Markov model for the remaining months of the 2 year time horizon, which was on average 19 months for patients with untreated CSA. Health states were defined in terms of disease activity score (DAS), using the number of tender joints, swollen joints, patient’s general health (measured on a visual analogue scale) and ESR level (systemic inflammation).^39^ Patients entered the Markov model in either remission (DAS≤1.6), low disease activity (DAS=1.6–2.4) or moderate/high disease activity (DAS>2.4).^39^

Patients who did not develop IA entered the Markov model from the start in the no IA health state. Figure 1 presents the decision tree combined with the Markov models for patients who developed IA and patients who did not develop IA for the watchful waiting strategy and the intervention strategies.

### Data sources

To populate the health economic model, we mainly used longitudinal data from four different data sources: the Leiden CSA cohort,^37^ the multicentre Rotterdam CSA cohort,^38^ the Leiden Early Arthritis clinic cohort^38 40^ and the TREAT EARLIER trial.^21^ More details about these data sources can be found in online supplemental file 2. The questionnaires to calculate medical consumption, productivity and quality of life were completed within all these data sources and more information about the patient characteristics can be found in Bour *et al*^41^ and van Mulligen *et al.*^23^

### Model inputs

Online supplemental file 3 tables S2a, S3–S7, provides an overview of all model input parameters, their mean estimates, their distributions for the probabilistic sensitivity analysis (PSA) and the source used to estimate the input parameter.

#### Modelled treatment effect

Three different types of treatment effects of timely identification and treatment were modelled simultaneously. The treatment effect was based on the TREAT EARLIER trial, which included 236 patients. first, the 2 year results from the TREAT EARLIER trial showed that treatment delayed the time to IA development by 3 months, as the mean time to IA development in the intervention group was 13 months and in the placebo group 10 months over the 2-year time period.^21^ Hence, in the base-case of the model patients with CSA who were treated developed IA on average after 8 months instead of 5 months because in the CSA cohorts the average time to IA development was 5 months.

Second, due to treatment in the CSA phase, a greater proportion of patients who did develop IA entered the Markov model in the low DAS health state rather than the moderate/high DAS health state. In the 2-year TREAT EARLIER trial results, 10%-point more patients had a low DAS at IA development and 10%-point fewer patients had a moderate/high DAS compared with the placebo group (online supplemental table S3).^21^

Thirdly, we modelled a positive treatment effect on EQ-5D utilities, medical consumption and productivity for all patients with subclinical inflammation on the imaging tests who were given treatment. This effect was obtained from the TREAT EARLIER trial, which found these effects in treated patients with CSA, irrespective of IA development.^23^ The effect of treating patients with CSA with subclinical inflammation was an improvement in utility of 0.0202 over a period of 2 years for autoantibody-positive patients and 0.0200 for autoantibody-negative patients, based on a subgroup analysis (ie, autoantibody status) of the TREAT EARLIER trial (online supplemental table S5).^23^ This treatment effect was the mean effect across all treated CSA patients in this trial irrespective of development of IA. For treated autoantibody-positive patients, the medical consumption costs were found to be 89% of the costs for untreated patients, and productivity costs were found to be 75%. For treated autoantibody-negative patients, these percentages were 79% and 95%, respectively (online supplemental table S7). As the TREAT EARLIER trial did not assess treatment effects on informal care, no effect on costs of informal care was applied in the health economic model.

#### Diagnostic values and probabilities

The probabilities of developing IA, the probabilities of having a positive imaging test and the test characteristics were based on the CSA cohorts of Leiden and Rotterdam. A positive conventional MRI scan was defined as having subclinical joint inflammation at one (or multiple) joint tissue(s) (synovitis, tenosynovitis and/or osteitis) in the hands or wrists, that is present in less than 5% of an age-matched healthy population,^42 43^ scored by two independent blinded readers.^44 45^ We assumed the same diagnostic value for the modified Dixon MRI, based on Boeren *et al*,^46^ because no data were available of modified Dixon MRI scans in patients with CSA. The diagnostic values of ultrasound were derived from a study by Orhndorf *et al*,^47^ which made a head-to-head comparison between conventional MRI and US, using MRI as the gold standard. The sensitivity and specificity of the imaging modalities can be found in online supplemental 3 tables S2a,b.

After developing IA, patients transitioned into the Markov Model. The initial distribution of patients over the health states was based on the early arthritis clinic data. The probability of starting in one of the initial health states was conditional on survival in the CSA phase. Mortality rates were based on the age-sex dependent mortality rates of 2023 from the Dutch Central Bureau of Statistics,^48^ assuming no increased mortality due to the relatively young age of the cohort. Autoantibody-positive and -negative patients were assumed to have the same demographic characteristics.

The transition probabilities between the health state in the Markov Model were based on the pooled mean and pooled SE of three time periods in which data were collected for the early arthritis clinic cohort: from the onset of IA to 4 months, from 4 to 12 months and from 12 to 24 months. The transition probabilities were conditional on survival and calculated separately for autoantibody-positive and -negative patients, see online supplemental table S4. We applied the same set of transition probabilities in every 4 month cycle.

#### QALYs

For patients who developed IA, we calculated the QALYs in the CSA phase based on a time-to-diagnosis analysis, explained in Bour *et al*.^41^ For patients with CSA with a positive imaging result who were treated, the development of IA was delayed by 3 months, based on the TREAT EARLIER trial.^21^ Hence, the time in the Markov model was either 19 months for the untreated patients or 16 months for the treated patients.

The EQ-5D utilities assigned to the health states were based on the early arthritis clinic cohort for patients who developed IA. For those who did not develop IA, the estimates were based on the CSA cohorts, see online supplemental table S5 for the input values. These input parameters were derived from linear mixed models, as explained in Bour *et al*.^41^

#### Medical consumption, productivity costs, and informal care costs

The same approach was used to estimate the medical consumption costs and the productivity costs over 2 years. To calculate the medical consumption costs and productivity costs in the CSA phase, the time-to-diagnosis analysis was used.^41^

The medical consumption costs and the productivity costs assigned to the health states were based on the early arthritis clinic cohort for patients who developed IA. For those who did not develop IA, the estimates were based on the CSA cohorts, see online supplemental table S7 for the input values. These input parameters were derived using the same linear mixed models, as explained in Bour *et al*.^41^ A one-time intervention cost was allocated to the intervention strategies for the imaging modality used and the intervention treatment costs, see online supplemental table S6.

Informal care costs during the CSA phase for those developing RA were estimated using the same type of statistic model as for productivity costs, healthcare costs and health effects. After IA progression (using data from early arthritis clinic cohort), as well as for individuals who did not develop IA (using data from CSA cohorts), informal care costs were estimated by calculating the mean informal care costs per health state, see online supplemental table S7 for these values.

### Model validation

Throughout the building process of the model, the model and its input parameters were validated by two rheumatologists (AHvM and PdJ) for clinical face validity. Model debugging was based on relevant tests of the TECHVER protocol.^49^ All relevant tests led to the expected results, see online supplemental file 4 table S8.

### Uncertainty analyses

To assess the impact of parameter uncertainty on cost-effectiveness results, we conducted a PSA based on 1000 iterations and a deterministic one-way sensitivity analysis (OWSA), from which we identified the 10 most influential parameters. Results of the PSA are shown in cost-effectiveness planes (CE-plane) and acceptability curves (CEAC).

In a value of information analysis, we estimated the expected value of perfect information (EVPI) by calculating the net benefit lost when a chosen strategy would not have been cost-effective (ie, the expected net benefit with perfect information minus the expected net benefit with the given information) and displayed this with expected loss curves (ELCs).^50^

### Scenario analyses

We conducted four scenario analyses and show results from a societal perspective.

Scenario 1: Adding a utility decrement for side-effects from treatment: Treatment with MTX could have adverse side effects (ie, gastrointestinal side effects, abnormal liver tests and increased infection risk).^51^ It is possible that these side effects are not fully captured by EQ-5D-5L. Therefore, we conducted a two-way sensitivity analysis varying both the proportion of treated patients with side-effects from 0 to 100% and the disutility due to treatment from 0 to 0.5 QALY over 2 years. In the base-case of this scenario analysis, a disutility of 0.05 QALY over 2 years was applied to 35% of the treated patients with CSA, based on the TREAT EARLIER trial.^21^

Scenario 2: Relative instead of absolute delay in time-to-IA-diagnosis: We explored how much results were impacted by a relative rather than an absolute delay in time-to-IA-diagnosis for treated patients. Instead of an absolute delay of 3 months, a relative delay of 1.7 months was applied. The source for the relative delay is the TREAT EARLIER trial.^21^

Scenario 3: No treatment effect on productivity, medical consumption and quality of life: We investigated how the results were affected by only modelling the effect of the diagnostic interventions in terms of the delay in time-to-IA-diagnosis and a higher number of patients starting the Markov Model in a lower DAS health state. We simultaneously left out the treatment effects on productivity, medical consumption and quality of life because these effects were mainly driven by patients with non-progressing CSA.^23^

Scenario 4: Only treatment effects for individuals developing IA: additional to scenario 3, we also investigated the cost-effectiveness when applying the trial-wide treatment effects on quality of life, medical consumption and productivity to individuals who developed IA only. This is highly conservative, since the trial-wide effect was estimated on all patients and not only those developing IA. We do not know the true treatment effect in this subgroup.

### Patient and public involvement

There was no patient or public involvement in this research.

## Results

### Cost-effectiveness results: base case

Table 2 provides an overview of the costs and effects, (incremental) NMB and ICER based on a full hierarchical analyses, for both autoantibody-positive and -negative patients from the societal perspective. For both subgroups, the modified Dixon MRI had the highest NMB and dominates the other strategies. However, in an analysis in which all diagnostic strategies were compared with watchful waiting (table 2, right side), they were all found to have a positive incremental NMB. However, the 95% CIs were wide and very limited effects in terms of QALY gains were observed. Autoantibody-positive patients showed cost-savings ranging from €1183 (-5348 to 2861) for the ultrasound strategy compared with watchful waiting to €1825 (-8041 to 3861) for the modified Dixon MRI strategy. Autoantibody-negative patients had incremental costs from €21 (-3843 to 3856) for the conventional MRI strategy compared with watchful waiting to -€409 (-4499 to 3681) for the modified Dixon MRI. QALYs gained ranged from 0.016 (-0.08 to 0.11) for autoantibody-positive patients to 0.006 (-0.04 to 0.06) for autoantibody-negative patients.

**Table 2:** Total costs and QALYs, NMB, incremental NMB compared with watchful waiting and the ICERs: societal perspective

Strategy	Full hierarchical analysis			Compared with watchful waiting	
	Costs (95% CI)	QALYs (95% CI)	NMB	iNMB versus ww	ICER versus ww
Autoantibody-positive					
Modified Dixon MRI	€32 631 (26 415 to 38 317)	1.439 (1.34 to 1.53)	€39 259	€2608	Dominant
Conventional MRI	€33 018 (27 256 to 38 780)	1.439 (1.35 to 1.52)	€38 887	€2239	Dominant
Ultrasound	€33 273 (29 108 to 37 317)	1.432 (1.36 to 1.50)	€38 286	€1610	Dominant
Watchful waiting	€34 456 (30 882 to 38 432)	1.423 (1.36 to 1.48)	€36 687	–	–
Autoantibody-negative					
Modified Dixon MRI	€29 151 (25 061 to 33 241)	1.451 (1.41 to 1.50)	€43 417	€748	Dominant
Ultrasound	€29 292 (25 711 to 32 888)	1.449 (1.41 to 1.50)	€43 172	€490	Dominant
Watchful waiting	€29 560 (26 262 to 32 991)	1.445 (1.40 to 1.50)	€42 670		
Conventional MRI	€29 581 (25 717 to 33 416)	1.451 (1.41 to 1.49)	€42 978	€308	€3101/QALY

ICER, incremental cost-effectiveness ratio; iNMB, incremental net monetary benefit; NMB, net monetary benefit; QALYs, quality adjusted life years; ww, watchful waiting.

Figure 2 shows that, for autoantibody-positive patients, the modified Dixon strategy was preferred. This strategy was cost-effective with a probability of 50% at a WTP threshold of €50 000/QALY. For autoantibody-negative patients, also the modified Dixon MRI strategy was cost-effective with a probability of 46% at a WTP threshold of €50 000/QALY.

**Figure 2:**
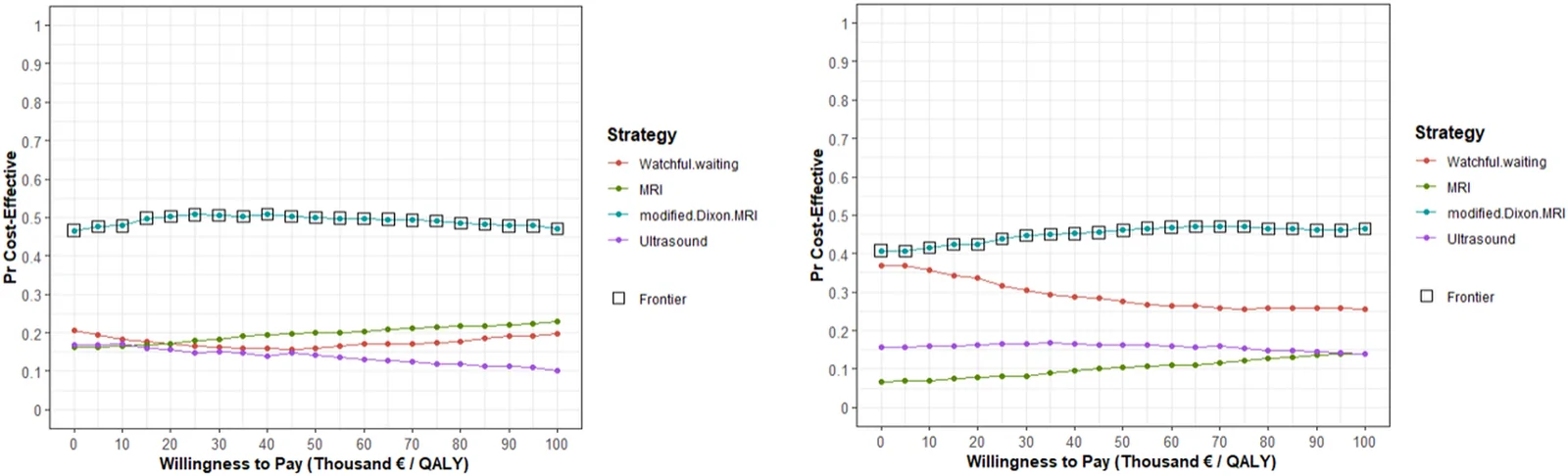
Cost-effectiveness acceptability curves (CEACs): societal perspective. QALY, quality-adjusted life years.

Figure 3 illustrates the CE-planes and CEACs for each intervention strategy compared with watchful waiting. Among autoantibody-positive patients, all intervention strategies were cost-effective, with a probability of 75% (MRI vs watchful waiting) to 80% (modified Dixon MRI vs watchful waiting) at a WTP threshold of €50 000/QALY. For autoantibody-negative patients, both QALY gains and cost-savings were small. However, all strategies were cost-effective with probabilities ranging from 56% (MRI vs watchful waiting) to 70% (modified Dixon MRI vs watchful waiting) at a WTP threshold of €50 000/QALY.

**Figure 3:**
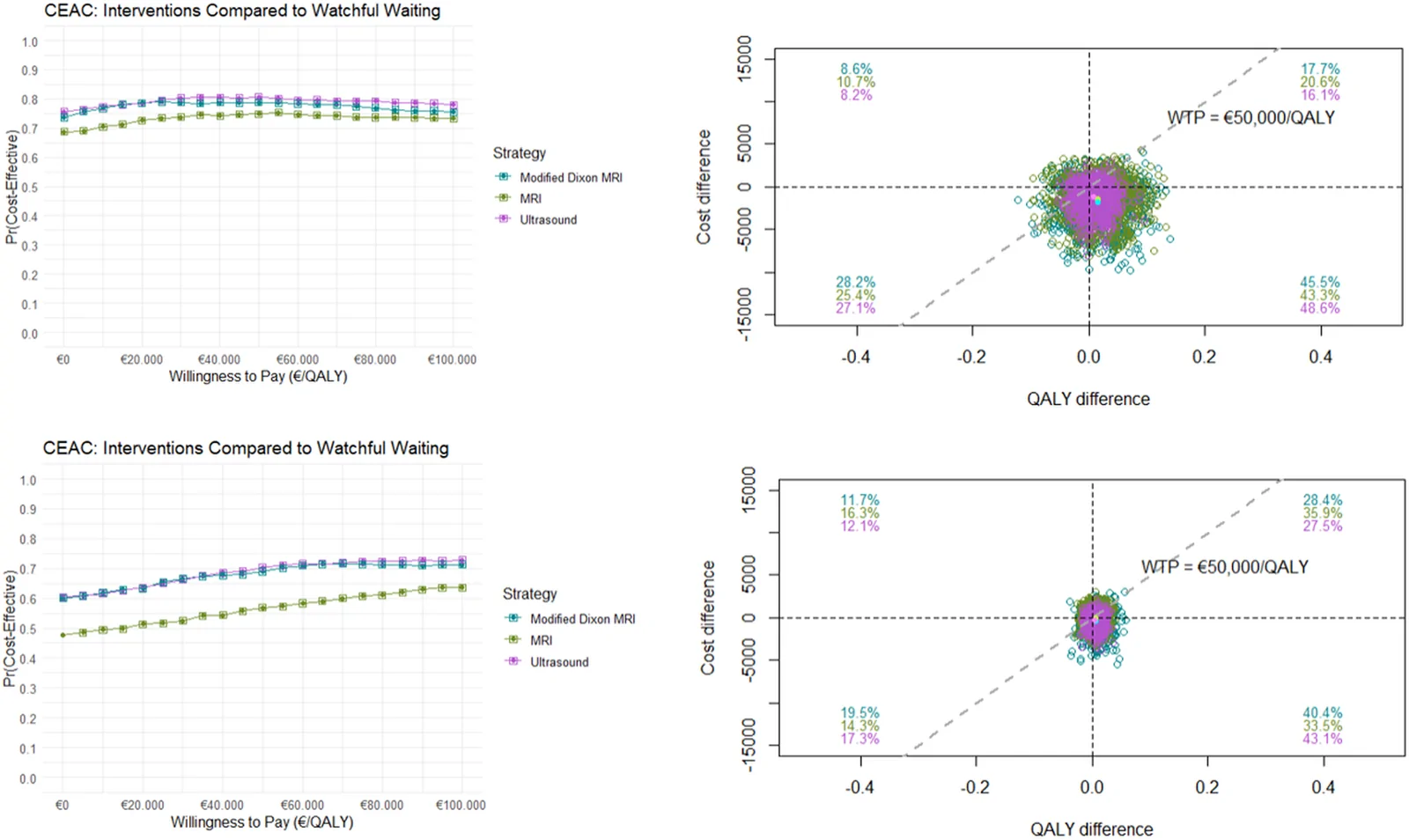
Cost-effectiveness acceptability curves and cost-effectiveness planes: interventions versus watchful waiting. CEAC, cost-effectiveness acceptability curve; CE-plane, cost-effectiveness plane; QALY, quality-adjusted life year; WTP, willingness to pay.

The main driver of cost differences between intervention and watchful waiting was productivity costs for autoantibody-positive patients, since treatment resulted in high productivity gains. So from the healthcare perspective, the differences between the intervention strategies and watchful waiting were less pronounced and cost-savings were limited, see online supplemental file 5 table S9. The CE-planes and CEACs from the healthcare perspective showed cost-effectiveness probabilities of 55% (MRI vs watchful waiting) to 60% (modified Dixon MRI vs watchful waiting) at a WTP threshold of €50 000/QALY for autoantibody-positive patients. For autoantibody-negative patients these were 50% (MRI vs watchful waiting) to 71% (modified Dixon MRI vs watchful waiting).

### One-way sensitivity analysis

The preferred imaging strategy was mainly sensitive to changes in diagnostic accuracy and less so to imaging modality costs.

The impact of the OWSA on the comparison between modified Dixon MRI versus watchful waiting is given in online supplemental file 6 table S10 for autoantibody-positive patients and in online supplemental file 7 table S11 for autoantibody-negative patient. For autoantibody-positive patients, only changes in the treatment effect on productivity and the treatment effect on QALYs had a considerable impact on the NMB. All other parameters resulted in positive NMBs over the full range of input values. The results of the OWSA for autoantibody-negative patients were similar.

### Value of information

Figure 4 presents the EVPI expressed with ELCs. The ELCs showed that for autoantibody-positive patients, the expected losses were higher than for the autoantibody-negative patients. This is due to the greater potential gains observed in autoantibody-positive patients. For autoantibody-negative patients, the expected losses for the modified Dixon MRI and ultrasound strategies were almost identical because of their similar NMBs.

**Figure 4:**
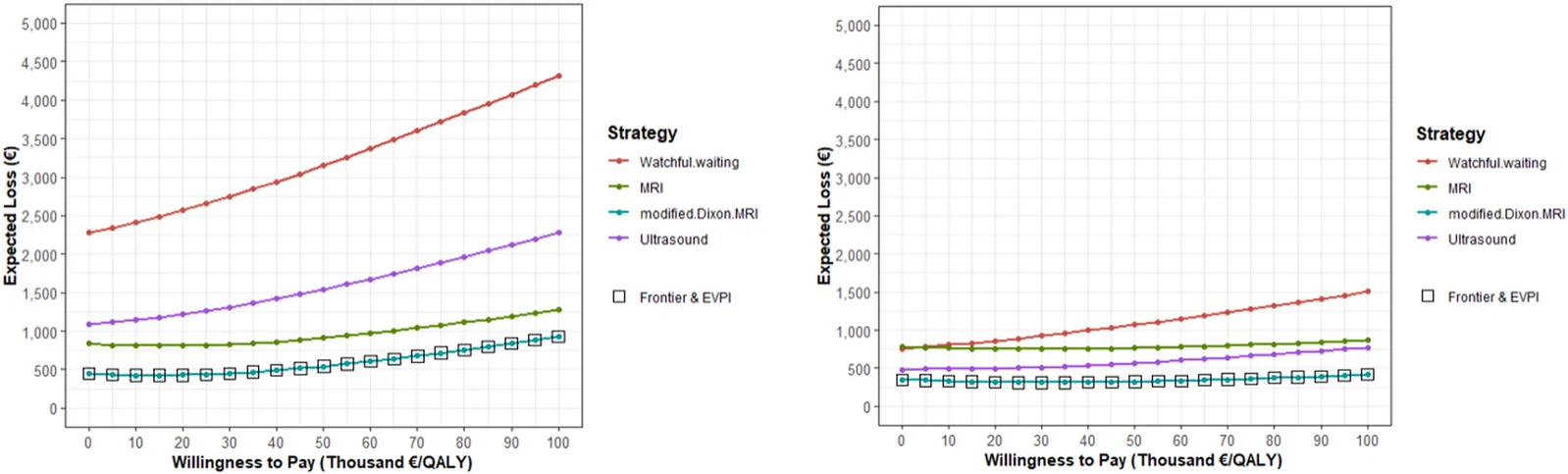
Expected loss curves: societal perspective. CEAC, cost-effectiveness acceptability curve; ELC, expected loss curve; EVPI, expected value of perfect information; Pr, probability; QALY, quality-adjusted life year.

A similar pattern was observed from a healthcare perspective, though the EVPIs were even closer to zero, as the potential gains were small, see online supplemental file 8 figure S4. The low EVPIs from this perspective suggest that the decision has minimal risk, as no intervention strategy clearly outperforms the others. As a result, the benefit of investing in additional research may not offset its costs.

### Scenario analyses

The tables and figures in online supplemental file 9 show the probabilistic outcomes from the scenario analyses. Scenario 1 showed that when 35% of the treated patients experienced a disutility of 0.05 over 2 years due to treatment-related side effects, the modified Dixon MRI strategy was still cost-effective for both autoantibody-positive and -negative patients at a WTP threshold of €50 000/QALY.

Additionally, we explored to what extent the effect of a varying utility decrement combined with the proportion of individuals facing that disutility affects the results. Figure 5 illustrates the combined effect of the magnitude of disutility experienced by treated patients and the proportion of patients affected.

**Figure 5:**
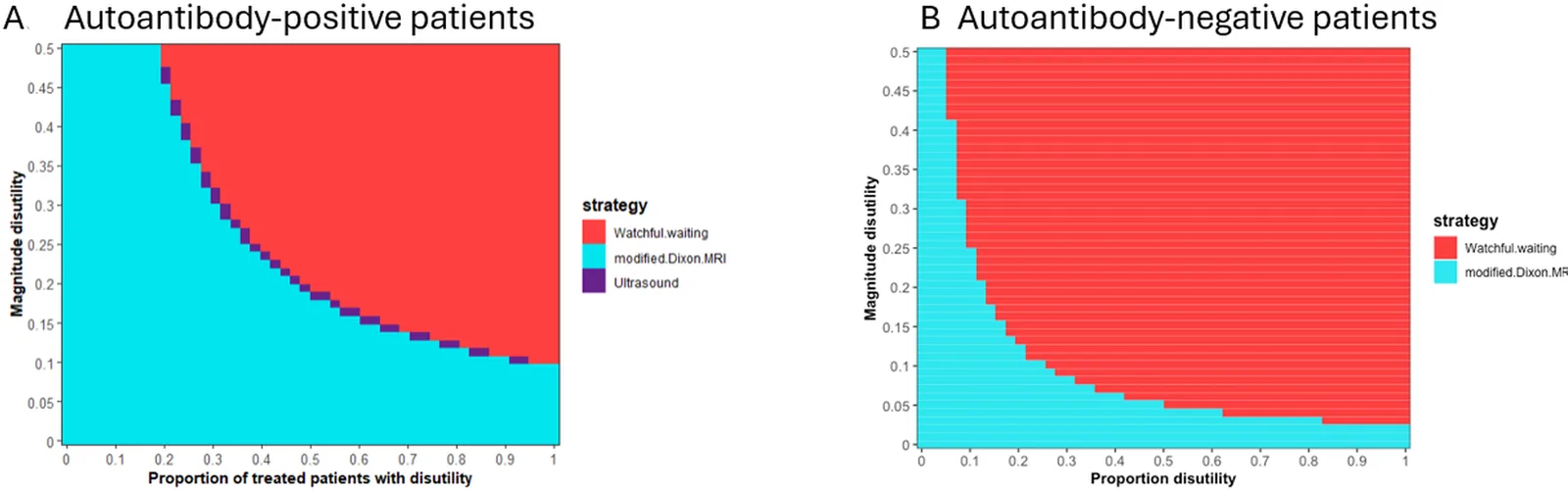
Two-way sensitivity analysis to explore the combined effect of magnitude and proportion of experienced disutility. *Proportion of treated patients with disutility=proportion of treated patients experiencing a disutility and magnitude disutility=magnitude of disutility faced by treated patients. Note: The colours indicate for which combinations of the input-parameters an intervention strategy is most cost-effective given a threshold of €50 000 per QALY. QALY, quality-adjusted life years.

For autoantibody-positive patients, the largest area of the graph is covered by modified Dixon MRI, indicating that this strategy remains cost-effective across a wide range of disutility magnitudes and proportions of patients affected. Only when the experienced disutility is high combined with a large proportion of patients affected, none of the intervention strategies is cost-effective. In contrast, for autoantibody-negative patients, watchful waiting is cost-effective for most combinations of the two model-input parameters. Only when the magnitude of disutility is low and/or affects a small proportion of patients, modified Dixon MRI is cost-effective.

The tables and figures in online supplemental file 9 showed that scenario 2, a relative delay in time-to-IA-diagnosis, had no effect on the outcomes of the CUA. In contrast, scenario 3 notably impacted cost-effectiveness results: when no improvement in health-related quality of life and no reduction in productivity loss and healthcare utilisation was assumed, none of the intervention strategies were cost-effective. However, scenario 4 showed that when the treatment effects on quality of life, medical consumption and productivity were only assumed for individuals developing IA, all intervention strategies were cost-effective compared with watchful waiting for autoantibody-positive patients. None of the intervention strategies were cost-effective for autoantibody-negative patients because only 9% of these individuals developed IA.

## Discussion

This study aimed to evaluate the cost-effectiveness of three diagnostic strategies—US, conventional MRI and modified Dixon MRI—combined with an early treatment strategy for patients with CSA, compared with watchful waiting. Our analyses, conducted from a societal perspective, demonstrated that diagnosing subclinical inflammation and initiating treatment with a single intramuscular glucocorticoid injection (120 mg) and a 1 year course of oral MTX for individuals with positive subclinical inflammation scores, had a 56% to 80% probability to be cost-effective compared with watchful waiting, depending on the imaging modality used. Among the strategies, modified Dixon MRI was preferred since it had the highest NMB and the highest probability to be cost-effective. For autoantibody-positive patients, this strategy had a probability of 50% of being cost-effective, and for autoantibody-negative patients, it had a probability of 46% at a WTP threshold of €50 000/QALY, comparing the four different strategies. From the healthcare perspective, while cost-savings and health benefits were less pronounced, all diagnostic strategies remained cost-effective. These results align with the trial-based CUA of the TREAT EARLIER trial.^23^ Nevertheless, if even very small QALY losses are considered unacceptable for any level of cost savings, the probability for any of the intervention strategies to be cost-effective would still be approximately 50%.

The OWSA confirmed these results, with most parameters resulting in positive NMBs across the tested input ranges. The only exceptions were the treatment effect on productivity and quality of life, which caused the diagnostic strategies not to be cost-effective at the most conservative input values.

Further validation was provided by the four scenario analyses, which generally supported our results. However, when a one-time disutility due to treatment-related side-effects was introduced, cost-effectiveness depended on both the proportion of patients affected and the magnitude of disutility, highlighting the importance of investigating this further. When only modelling the effect of the diagnostic interventions in terms of the delay in time-to-IA-diagnosis and simultaneously leaving out treatment effects on quality of life, productivity, and medical consumption, none of the intervention strategies remained cost-effective. Additionally, when these treatment effects were only assigned to individuals who developed IA, the intervention strategies were cost-effective compared with watchful waiting for autoantibody-positive patients only. While these two scenarios probably underestimate the treatment effect, given the TREAT EARLIER trial demonstrated positive effects on quality of life, medical consumption and productivity, caution is needed, as these analyses were done in the total trial population and recent data has shown that the total CSA population is heterogeneous in pathophysiology and risk of RA.^52–54^ This appears to blur treatment effects for certain subgroups. Patients with ACPA-negative CSA at increased risk for RA, as determined by the presence of extensive subclinical joint inflammation on MRI, were found to have the highest treatment effectiveness, with benefits that reduced both RA progression and disease burden.^52 55^ Patients who were ACPA-positive and at increased risk for RA had a temporary benefit for 2 years, but not for 5 years, while patients who were retrospectively at low risk for RA had no benefit at all.^52 55^ This could mean that in our analysis the overall treatment effect may have been diluted by including a substantial proportion of autoantibody-negative low risk individuals (ie, patients with low subclinical inflammation scores). Hence, targeting high-risk subgroups may improve clinical and economic benefits of early intervention. Importantly, MRI was important for risk prediction; the number of locations of subclinical inflammation, and presence of the metacarpophalangeal-extensor tenosynovitis^52 56^ were crucial predictors and these can only be accurately detected using MRI and not through US.

Future research should focus on refining risk stratification. A recently published paper from the ACR/EURLAR identified risk stratification criteria for individuals with arthralgia at risk of developing RA.^57^ The identified risk stratification criteria were clinical variables (ie, morning stiffness, patient reported swelling and difficulty making a fist), serologic markers (ie, CRP, RF and ACPA) and MRI-detected tenosynovitis, with an area under the curve of 0.86.^57^ We have to evaluate outcome measures within these risk-strata to improve the precision and impact of early treatment strategies.

### Strengths and limitations

The health economic model that we created is the first to simulate the effects of early diagnosis and treatment in the CSA phase. It is also unique because of its integration of data from four different data sources that reflected the periods before and after the onset of IA. Moreover, we had access to patient-level data from the TREAT EARLIER trial that enabled the estimation of specific input parameters.

However, the model also has some limitations, primarily related to the input parameters. First, the lack of long-term follow-up data in the TREAT EARLIER trial for patients with CSA who developed IA potentially led to an underestimation of the treatment effects post-IA diagnosis. In the model, we accounted only for a 10%-point increase in patients starting in the low DAS health state, without adjusting transition probabilities after IA development. Due to data constraints and uncertainty in long-term extrapolation, we limited the analysis to a 2 year time horizon. Besides, no treatment effect was assumed after IA development; therefore, differences in costs and QALYs would remain unchanged with a lifetime horizon. This is a conservative assumption which potentially underestimates the lifetime cost-effectiveness results. Since the aim of our study was to assess the cost-effectiveness of early diagnostic strategies combined with an early treatment strategy, this time horizon captured the relevant outcomes. Second, the diagnostic values of ultrasound were likely underestimated because those were estimated based on a head-to-head comparison with conventional MRI, without using IA development as the endpoint.^47^ In this comparison, conventional MRI was considered the gold standard, which may have led to underestimation of ultrasound performance.

Third, the diagnostic values of modified Dixon MRI were perhaps under- or over-estimated, as we assumed they were identical to conventional MRI based on a comparative study between conventional MRI and modified Dixon MRI in an early arthritis cohort.^46^ However, the diagnostic accuracy was not 100% the same between these two imaging techniques; although a high correlation is indeed demonstrated in the stage of RA, such information from the risk stage of CSA is still lacking. However, this would not have affected the finding that all strategies outperformed watchful waiting.

Fourth, the treatment effect observed in patients with subclinical inflammation based on MRI was generalised to other imaging modalities. However, the effect may have differed if subclinical inflammation was detected using US or modified Dixon MRI.

Lastly, a timely recognition of CSA is pivotal for the implementation of the diagnostic and therapeutic strategy studied. The Leiden University Medical Centre has focused on this task for the past two decades.^58^ This may hamper the generalisability of these results to other rheumatology centres. They would first have to optimise the referral process to recognise patients at the risk stage of CSA, rather than at the time of RA diagnosis, to be able to implement an early treatment strategy.

### Implications and recommendations

Treatment can have a positive impact on patients with CSA who develop IA as well as those who do not develop IA, particularly in terms of productivity. However, shared decision-making between patient and physician is essential, given the potential side effects of MTX.^51^ To minimise overtreatment, identifying high-risk subgroups and refining targeted treatment is important.

Several studies have shown that patients at risk of developing IA are willing to take preventive treatment.^59–61^ One study showed that a high proportion of patients with CSA, especially the ones who did not develop IA, would recommend treatment in the CSA phase to others.^59^ Another study showed that individuals with arthralgia at-risk of developing IA stated more often they would pursue predictive testing (50% vs 10%), consider preventive medication (38% vs 10%) and adopt lifestyle changes (83% vs 20%) compared to asymptomatic individuals at risk.^60^ The latter suggests that patients at risk of developing RA may also consider non-pharmaceutical interventions, such as lifestyle changes (ie, stop smoking, healthy weight, physical activity, dental hygiene, vitamin D and high intake of omega-3-fatty acids),^61^ although concrete evidence of their effectiveness is scarce.

Our findings suggest that modified Dixon MRI or US, combined with treatment, are promising strategies for managing CSA, considering the limitations. Although conventional MRI is also cost-effective compared with watchful waiting, its higher costs, limited availability and lengthy protocol make it less practical.^18^ However, modified Dixon MRI as well as ultrasound need further research to validate their diagnostic value and associated treatment effects for patients with CSA with subclinical inflammation before implementation in clinical practice.

In conclusion, imaging-guided detection of patients with high-risk CSA combined with treatment with a single intramuscular glucocorticoid injection (120 mg) and a 1 year course of oral MTX may be cost-effective from the societal perspective. However, the results are less pronounced for the total autoantibody-negative CSA population and the health benefits on this population as a whole seem rather small. From a healthcare perspective, the cost-effectiveness of the diagnostic strategies remains uncertain. Our findings support initiating treatment in the CSA phase guided by patient characteristics based on autoantibodies and imaging-detected subclinical inflammation. Collecting real-world evidence on treatment outcomes is needed to confirm our findings.

## Supplementary material

10.1136/bmjopen-2026-118495 online supplemental file 1

## Data Availability

Data are available upon reasonable request.
