# Supplementary material for: Imaging-guided treatment strategy compared to standard care in at-risk individuals for rheumatoid arthritis: a cost-utility analysis
Source: BMJ Open. 2026 Oct 21;16(10):e118495. doi: 10.1136/bmjopen-2026-118495 (PMC13691768; doi:10.1136/bmjopen-2026-118495)
Supplement: Supplementary file 1 [file bmjopen-2026-118495.reviewer_comments.pdf]

PEER REVIEW HISTORY

BMJ Open publishes all reviews undertaken for accepted manuscripts. Reviewers are asked to complete a checklist review form and are provided with free text boxes to elaborate on their assessment. These free text comments are reproduced below.

ARTICLE DETAILS

Title (Provisional)

An imaging-guided treatment strategy compared to standard care in at-risk individuals for rheumatoid arthritis: A cost-utility analysis

Authors

Bour, Sterre; Van Mulligen, Elise; Goossens, Lucas; de Jong, Pascal Hendrik Pieter; van der Helm - van Mil, Annette; Rutten-van Molken, Maureen

---

VERSION 1 - REVIEW

**Reviewer**	**1**
**Name**	**Muller, Dirk**
**Affiliation**	**University Hospital Cologne, Institute of Health Economics and Clinical Epidemiology**
**Date**	**18-Mar-2026**
**COI**	**None**

The cost-utility analysis submitted by Bour et al. evaluates imaging-guided treatment strategy compared to SC in at-risk individuals for rheumatoid arthritis. While the manuscript provided sufficient information for most areas addressed in the added CHEERs-checklist, concerns remain for the 'modelled treatment effect' (subchapter 2.4.1) and costs which were based on the TREAT EARLIER trial. According to that trial, early treatment "did not prevent the development of clinical arthritis, but modified the disease course". The present CUA now claims significant QALY-gains (0.005 to 0.015), resulting in a high probability of cost-effectiveness and prompting decision maker to initiate early treatment. According to the authors, they "modelled a positive treatment effect on EQ-5D utilities… for all patients with subclinical inflammation on the imaging tests who were given treatment. This effect was obtained from the TREAT EARLIER trial" where QoL via EQ-5D was assessed only as an explorative endpoint. This should be clearly mentioned and the following points would be required to have outlined with more transparency:

1) Data on utility is presented in table S5. If I understand it correctly, all of this is unpublished data, including the treatment effect below in the table. Or refer the values cited as CSA cohort Leiden & Rotterdam to Boer et al (54)?

2) With regard to the first effect in 2.4.1 a 3-month-delay to IA due to treatment was applied. I looked for this information in the Earlier-trial and found it confirmed for a very small subgroup analysis (figure 3 B in Krijbolder et al., Lancet 2022). Is this the place within the TREAT EARLIER trial you referred to and was this assumption tested in a sensitivity analysis? Please clarify.

3) With regard to the third treatment effect please mention what the extent of this effect is based on (e.g., the number of patients who responded to the EQ-5D-questionnaire).

4) The same should be for data on costs.

Minor concerns:

Abstract: could be shortened in the sections ‚Objective‘ and ‘Design’ (e.g., cost instruments could be deleted) but information about probabilistic sensitivity analysis should be added in the ‘Design’-section.

Abstract (‘Results’): line 37-39 already refers to a result of the PSA, while the last sentence begins with ‘Sensitivity analysis confirmed...” Please clarify.

Introduction, last sentence: please mention the targeted patient group in the study question.

Page 7, section 2.1.4: please add the reason mentioned on page 4 for the two-year time horizon (which differs from the reason provided here). Taking a longer time horizon or comparing different time horizons would have allowed to receive a more holistic picture of the cost-effectiveness. Have any efforts been made to find appropriate evidence to extent the model?

Page 7, section 2.1.4, line 10/11: the cited reference (41) refers to the iMTA-Manual without clarifying who and how many responded to the questionnaire.

Section 2.4.4., first sentence: Do you mean that medical consumption, productivity costs, and informal care costs were taken from the TREAT EARLIER-trial and the CSA cohort? Please clarify.

**Reviewer**	**2**
**Name**	**Auguste, Peter**
**Affiliation**	**University of Warwick Warwick Medical School**
**Date**	**30-Mar-2026**
**COI**	**None**

Introduction

The introduction is well structured but could benefit with background information about rheumatoid arthritis from a Dutch perspective.

Methods

Authors have provided details of the conduct of the model based economic analysis to include study population, location and setting, model structure, and planned analysis, which are in line with stated best practice guidelines. Here, I have outlined a few points:

It would have been helpful to know early on the starting age of the population, and male and female split.

Additionally, what assumption is being made about the test accuracy of using ultrasound, conventional MRI and modified Dixon MRI? Are the authors assuming that test accuracy is the same across all three imaging modalities. If so, this should be stated and referenced. If not, how are errors (false negative and false positive) being included in the model? The structure provided does not show how these errors are captured and their pathways.

The authors used a hybrid structure comprising decision tree and Markov nodes/models. I think that these two phases of the model need further explanation. For example, on pg 6 line 45, 'Patients remained in the decision tree until they developed IA'. On pg 7 line 43, 'Patients who did not developed IA, entered the Markov model from the start in the no IA health state.' These statements appear inconsistent on what happened to people who did not develop IA.

The authors should be clear that there are two Markov models used, for people who developed IA and for people who did not develop IA. It would have been simpler to have one Markov model, which included those people who did not develop IA.

It is unclear where some of the inputs were obtained or derived. For example, patients who developed IA, entered the Markov model for the remaining months of the two-year time horizon. What does remaining months mean and how was this derived?

Have the authors applied a half-cycle correction?

Information is provided on the transition probabilities included in the model. Based on the Markov structure, people can transition to the dead state, but the transition matrices presented in Table S4 do not reflect this. What values are used for people transitioning to the dead state? Is there an increased risk of death for people who have IA compared to no IA.

Given that transition matrices (Table S4) are being used I would have expected there to be correlation between the parameters and hence the use of Cholesky decomposition in the probabilistic sensitivity analysis (PSA). Unless there is no correlation between the matrices (huge assumption), I am not convinced that the full uncertainty in the transition matrix is being captured.

Is the decision tree structure a reflection of the hybrid structure? If so, it might have been more appropriate to include a structure with the Markov nodes.

Results

Authors reported deterministic and probabilistic sensitivity analyses results, which showed that all interventions were most costly and expected to yield more QALYs than the watchful waiting strategy. From table 2, the QALYs yielded appear higher than expected given that the time horizon is only two years.

The authors stated that a 'full hierarchical' analysis had been undertaken. I take this to mean full incremental analysis. However, I am not convinced that these results are reported, and this is confirmed by the results reported in Table S9. The ICERs reported are pairwise ICERs for the comparisons between diagnostic imaging versus watchful waiting. Hence, there is no comparison between the diagnostic imaging strategies.

It would have been useful to see results in terms of cost per diagnostic error avoided along with cost per life year and cost per QALY results.

The authors stated that modified Dixon strategy was cost-effective with a probability of 49% at a WTP threshold of €50,000/QALY. However, it might be more appropriate to state that modified Dixon when compared to watchful waiting had a 0.49 probability of being cost-effective at a WTP threshold of €50,000/QALY.

For people unfamiliar with presenting results in terms of NMB, the authors should state how these should be interpreted.

Results form the PSA showed that a considerable number of iterations were in the southwest quadrant indicating that the intervention is less costly and less effective than watchful waiting; however, there is no discussion about that in the manuscript.

General comments

The manuscript contains lots of acronyms. Please can the authors consider not using non-standard acronyms. Examples, IA, CSA, UA, US, EAC

VERSION 1 - AUTHOR RESPONSE

Reviewer: 1
Dr. Dirk Muller, University Hospital Cologne
Comments to the Author:
The cost-utility analysis submitted by Bour et al. evaluates imaging-guided treatment strategy compared to SC in at-risk individuals for rheumatoid arthritis. While the manuscript provided sufficient information for most areas addressed in the added CHEERs-checklist, concerns remain for the 'modelled treatment effect' (subchapter 2.4.1) and costs which were based on the TREAT EARLIER trial. According to that trial, early treatment "did not prevent the development of clinical arthritis, but modified the disease course". The present CUA now claims significant QALY-gains (0.005 to 0.015), resulting in a high probability of cost-effectiveness and prompting decision maker to initiate early treatment. According to the authors, they "modelled a positive treatment effect on EQ-5D utilities... for all patients with subclinical inflammation on the imaging

tests who were given treatment. This effect was obtained from the TREAT EARLIER trial" where QoL via EQ-5D was assessed only as an explorative endpoint. This should be clearly mentioned and the following points would be required to have outlined with more transparency:

- Our point estimates for QALY gains varied between 0.005 and 0.015, depending on the strategy, but all had wide confidence intervals, which included positive and negative values. The CIs can be calculated from the 95% CIs in table 2 and are added to the results and in the abstract of the manuscript.

1. Data on utility is presented in table S5. If I understand it correctly, all of this is unpublished data, including the treatment effect below in the table. Or refer the values cited as CSA cohort Leiden & Rotterdam to Boer et al (54)?

The values in table S5 are based on unpublished data. Since we had access to the TREAT EARLIER data the treatment effect estimate on EQ-5D scores was stratified based on autoantibody status instead of for the whole trial population. Therefore, these numbers cannot be directly found in the published article of van Mulligen et al [23]. We do explain this in paragraphs 2.4.3 and 2.4.5 of the methods section. We clarified it by adding a few sentences to page 10 of the manuscript:
The effect of treating CSA patients with subclinical inflammation was an improvement in utility of 0.0202 over a period of two years for autoantibody-positive patients and 0.0200 for autoantibody-negative patients, based on <u>a subgroup analysis of the</u> TREAT EARLIER trial (lower limit scenario) <u>based on the same method as for the full trial population</u> [23]. This treatment effect was the mean effect across all treated CSA patients in this trial irrespective of development of IA.

Treatment effects were based on the CUA of the TREAT EARLIER trial (lower limit scenario) <u>but additional subgroup analyses were performed</u> [23]. For treated autoantibody-positive patients, the medical consumption costs were found to be 89% of the costs for untreated patients, and productivity costs were found to be 75%. For treated autoantibody-negative patients, these percentages were 79% and 95%, respectively.

We also had access to the patient-level data of these cohorts, so therefore we did additional analyses on EQ-5D utility scores and costs. However, for the estimation of the EQ-5D utility scores the same data were used from the CSA cohorts and early arthritis clinic cohort as in Bour et al (49). *<u>We added a reference to Bour et al in table S5, page 34</u>*.

2) With regard to the first effect in 2.4.1 a 3-month-delay to IA due to treatment was applied. I looked for this information in the Earlier-trial and found it confirmed for a

very small subgroup analysis (figure 3 B in Krijbolder et al., Lancet 2022). Is this the place within the TREAT EARLIER trial you referred to and was this assumption tested in a sensitivity analysis? Please clarify.

The 3-month-delay was not based on the subgroup analysis performed in figure 3B but on the analysis in figure 3A. Figure 3A shows the number of patients developing RA at each timepoint for the whole trial population. It was calculated as the difference in mean time to IA development between the treatment group (13 months) and the placebo group (10 monhts) over the 2-year time period.

We did perform a scenario analysis assuming a delay of 1.7 months, as individuals in the CSA cohorts Rotterdam and Leiden developed IA quicker (i.e., in 5 months) compared to individuals in the TREAT EARLIER trial. This assumption did not lead to different results of the CUA.

3) With regard to the third treatment effect please mention what the extent of this effect is based on (e.g., the number of patients who responded to the EQ-5D-questionnaire).

The treatment effect was based on the TREAT EARLIER trial, which included 236 patients. The treatment group consisted of 119 patients and the placebo group of 117 patients and all of those patients responded to the EQ-5D questionnaire. We stratified based on ACPA and RF positivity. 39 patients were autoantibody positive in the treatment arm and 38 in the placebo group. We clarified the number of patient who participated in the TREAT EARLIER trial and added it to paragraph 2.4.1 page 9: *"The treatment effect was based on the TREAT EARLIER trial, which included 236 patients."*

The treatment effect was based on the trial-based cost-effectiveness study of the TREAT EARLIER trial (van Mulligen [23]). Those results were based on the full trial population. However, we stratified based on ACPA/RF positivity, therefore our inputs were different from the published treatment effect. In paragraph 2.3 Data sources, we do refer to van Mulligen et al(2024) now. Page 9: *"The questionnaires to calculate medical consumption, productivity, and quality of life were completed within all these data sources, and more information about the patient characteristics can be found in Bour et al (2025)[49] and van Mulligen et al (2024)[23]"*

4) The same should be for data on costs.
The same data source was used for costs (see response to question 3).

Minor concerns:
5. Abstract: could be shortened in the sections ‚Objective' and 'Design' (e.g., cost

instruments could be deleted) but information about probabilistic sensitivity analysis should be added in the 'Design'-section.

We made the changes in the abstract on page 2 of the manuscript.

*"**Objective** Some individuals present with symptoms before RA develops, and trials suggest that early treatment during this at-risk phase may be effective. This study evaluated the cost-utility of three imaging strategies—conventional MRI, modified Dixon MRI, and ultrasound —combined with early treatment compared with watchful waiting in individuals at risk of RA, stratified by autoantibody status."*

*"**Design:** A decision tree combined with a Markov model was developed to estimate costs and quality-adjusted life years (QALYs) over a 2-year time horizon. Model inputs were derived from the TREAT EARLIER trial for treated patients at risk of developing RA, Clinically Suspect Arthralgia (CSA) cohorts for untreated at-risk patients, and the early arthritis clinic for patients with undifferentiated arthritis or RA. Cost-utility analyses were conducted from both the societal- and healthcare perspectives. Probabilistic and deterministic sensitivity analyses were conducted to assess the uncertainty of the results. Besides, several scenarios were explored."*

6. Abstract ('Results'): line 37-39 already refers to a result of the PSA, while the last sentence begins with 'Sensitivity analysis confirmed…" Please clarify.

We clarified this in the abstract by referring to the results of the deterministic sensitivity analyses. Page 2, Results: *"Deterministic sensitivity analysis confirmed robustness, but it depended on treatment effects, and treatment disutility."*

7. Introduction, last sentence: please mention the targeted patient group in the study question.

We added this to the last sentence of the introduction, page 4: *"The aim of this study is to assess the cost-utility of three imaging strategies – conventional MRI, modified Dixon MRI, and ultrasound – followed by early-treatment of patients with a positive imaging result, compared to watchful waiting, for individuals at risk of developing RA."*

8. Page 7, section 2.1.4: please add the reason mentioned on page 4 for the two-year time horizon (which differs from the reason provided here). Taking a longer time horizon or comparing different time horizons would have allowed to receive a more holistic picture of the cost-effectiveness. Have any efforts been made to find appropriate evidence to extent the model?

We did not apply a longer time horizon because no differences between the intervention strategies and watchful waiting were assumed after 2 years. As a result, extending the time horizon would not change outcomes in the current model. In the current model we did not assume differences after progression to IA except that 10%-points more patients

initially entered the low DAS health state. Since patients generally move quickly towards remission after treatment initiation, this effect diminishes.

At the time of writing this manuscript, no evidence was available supporting treatment effects beyond 2-years. It might be a conservative assumption, so we added this to the discussion (page 15): *"Besides, no treatment effect was assumed after IA development, therefore differences in costs and QALYs would remain unchanged with a lifetime horizon. This is a conservative assumption which potentially underestimates the lifetime cost-effectiveness results"*

9. Page 7, section 2.1.4, line 10/11: the cited reference (41) refers to the iMTA-Manual without clarifying who and how many responded to the questionnaire.

Individuals in the CSA cohorts, the early arthritis clinic cohort, and in the TREAT EARLIER trial responded to this questionnaire. 507 patients in the CSA cohorts (369 from the Leiden CSA cohort and 138 from the multicenter Rotterdam cohort), 282 in the early arthritis clinic cohort and 236 in the TREAT EARLIER trial, responded to this questionnaire.

To clarify we referred to the studies using these patient populations in paragraph 2.3 Data source (page 8): *"The questionnaires to calculate medical consumption, productivity, and quality of life were completed within all these data sources, and more information can be found in Bour et al (2025)[49] and van Mulligen et al (2024)[22]"*

10. Section 2.4.4., first sentence: Do you mean that medical consumption, productivity costs, and informal care costs were taken from the TREAT EARLIER-trial and the CSA cohort? Please clarify.

The input parameters for medical consumption costs, productivity costs, and informal care costs were based on the CSA cohorts (Leiden and Rotterdam) for the CSA phase and the early arthritis clinic cohort for the phase after IA development. The treatment effects were based on the TREAT EARLIER trial and those were applied to the CSA population.

To clarify this we added an explanation to paragraph 2.4.4 (page 9): *"To calculate the medical consumption costs and productivity costs in the CSA phase the time-to-diagnosis analysis was used[48]. The medical consumption costs and the productivity costs assigned to the health states were based on the early arthritis clinic cohort for patients who developed IA. For those who did not develop IA, the estimates were based on the CSA cohorts. These input parameters were derived using the same linear mixed models, as explained in Bour et al[48]."*

Reviewer: 2
Mr. Peter Auguste, University of Warwick Warwick Medical School
Comments to the Author:
Introduction
1. The introduction is well structured but could benefit with background information about rheumatoid arthritis from a Dutch perspective.

We included a sentence in the introduction about the incidence of RA in the Netherlands. Page 4: *“with around 17,400 new cases annually in the Netherlands[3]”.*

Methods
Authors have provided details of the conduct of the model based economic analysis to include study population, location and setting, model structure, and planned analysis, which are in line with stated best practice guidelines. Here, I have outlined a few points:

2. It would have been helpful to know early on the starting age of the population, and male and female split.
We added this to paragraph 2.1.1 Study population, setting and location and deleted this from paragraph 2.4.2. Page 5: *“Individuals at risk of inflammatory arthritis (IA, in this case being either undifferentiated arthritis or RA) either developed IA within 2 years or they did not. The mean age of individuals with CSA who did not develop IA was 44 and the male/female ratio was 21%/79%. Those who developed IA had a mean age of 48 and a male/female ratio of 28%/72%.”*

3. Additionally, what assumption is being made about the test accuracy of using ultrasound, conventional MRI and modified Dixon MRI? Are the authors assuming that test accuracy is the same across all three imaging modalities. If so, this should be stated and referenced. If not, how are errors (false negative and false positive) being included in the model? The structure provided does not show how these errors are captured and their pathways.

To clarify which diagnostic values were used we added where the sensitivity and specificity can be found (page 10): *The sensitivity and specificity of the imaging modalities can be found in supplemental file 3, table S2a*.
We explained in paragraph 2.4.2 Diagnostic values and probabilities (pages 8 and 9). We did not assume the same test accuracy for ultrasound. We used the study of Ohrndorf et al[54] which made a head-to-head comparison between ultrasound and conventional MRI. We did assume the same test accuracy for Modified Dixon MRI as of conventional MRI. We

**Imaging test**	Autoantibody positive patients		Autoantibody negative patients	
	Sensitivity	Specificity	Sensitivity	Specificity

Ultrasound	0.41	0.78	0.34	0.79
Conventional MRI	0.83	0.68	0.65	0.70
Modified Dixon MRI	0.83	0.68	0.65	0.70

The false positives, received treatment and based on the TREAT EARLIER trial also false positives did show a treatment effect. Therefore, all positive patients received the same treatment effect in the model because we could not stratify based on outcome (IA or no IA development). This is clarified on page 9: *Thirdly, we modelled a positive treatment effect on EQ-5D utilities and productivity for all patients with subclinical inflammation on the imaging tests who were given treatment. This effect was obtained from the TREAT EARLIER trial, which found these effects in treated CSA patients, irrespective of IA development[23].*

False negatives, did not receive early treatment and therefore were treated similarly as in the watchful waiting strategy. So those patients did not have a treatment effect (see the proposed new figure 1).

Since "false positives" also experienced a treatment effect, they might not really be false positives, they just did not develop the endpoint of interest. Therefore, we decided to not incorporate this explicit grouping.

4. The authors used a hybrid structure comprising decision tree and Markov nodes/models. I think that these two phases of the model need further explanation. For example, on pg 6 line 45, 'Patients remained in the decision tree until they developed IA'. On pg 7 line 43, 'Patients who did not developed IA, entered the Markov model from the start in the no IA health state.' These statements appear inconsistent on what happened to people who did not develop IA.

To clarify points 4, 5 and 9 from the reviewer, we developed a new figure 1 with a footnote to explain the model structure more clearly. In response to this point of the reviewer we changed the sentence on page 7: *"Patients who would develop IA remained in the decision tree until they progressed to IA."*

Figure 1. Schematic overview of the model

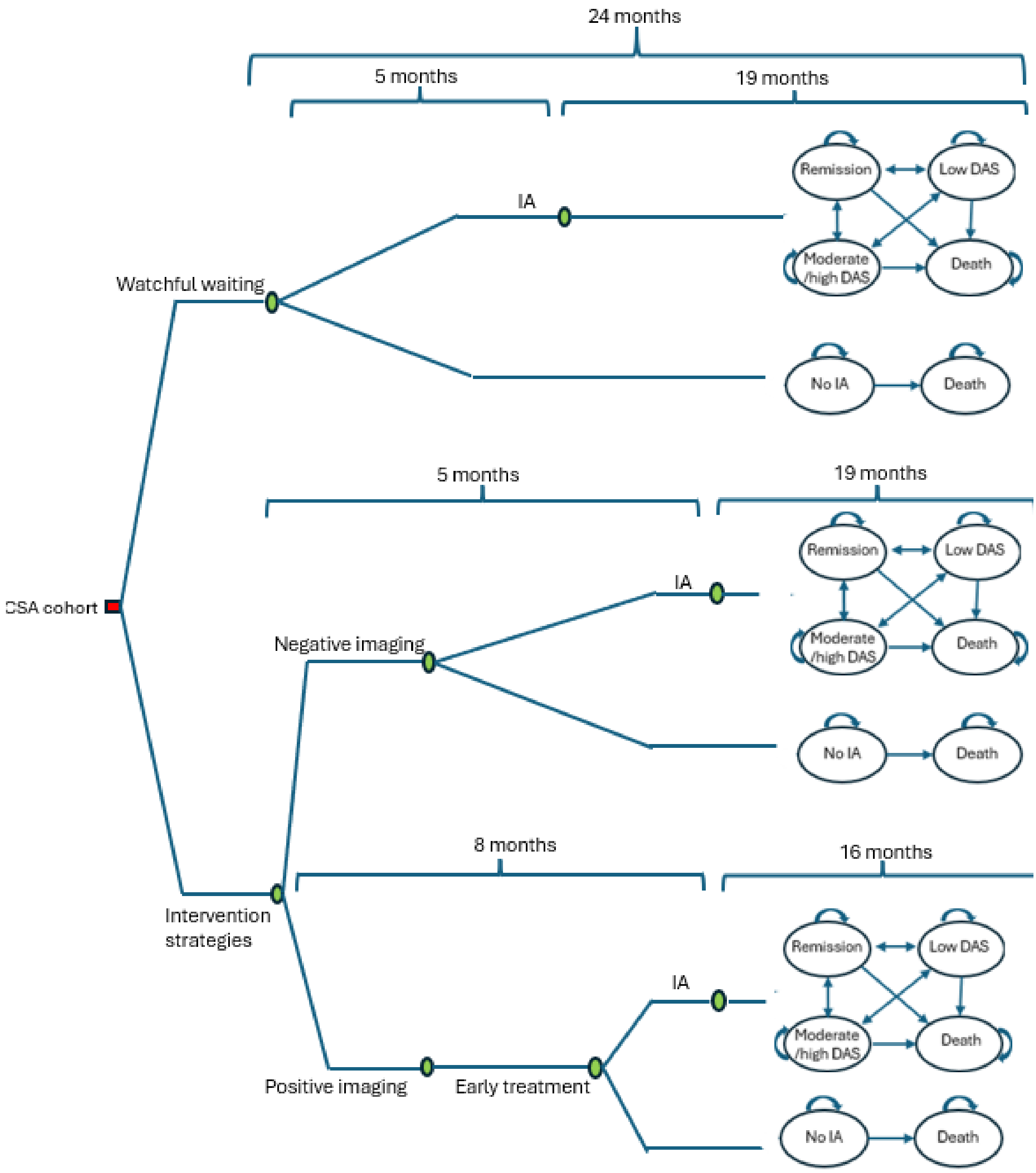

Note: *The model used a total time horizon of 24 months. Patients entered a decision tree followed by a Markov model, with the timing of transition depending on the strategy and whether inflammatory arthritis (IA) developed. In the watchful waiting strategy, patients who developed IA did so after an average of 5 months. These individuals remained in the decision tree for 5 months and then entered the Markov model for the remaining 19 months. Upon entry, they could transition into one of four health states: remission, low disease activity (DAS), moderate/high DAS, or death. Patients who did not develop IA entered the Markov model immediately in the "No IA" health state for 24 months and were only subject to background mortality. In the intervention strategies, patients with a positive imaging result who received early treatment experienced a delay in IA onset of 3 months compared to untreated patients (see paragraph 2.4.1). As a result, IA developed after an average of 8 months, after which patients entered the Markov model for the*

remaining 16 months. Patients with a negative imaging result followed the same pathway as in the watchful waiting strategy, with an average time to IA development of 5 months if IA occurred, or immediate entry into the "No IA" state otherwise.

5. The authors should be clear that there are two Markov models used, for people who developed IA and for people who did not develop IA. It would have been simpler to have one Markov model, which included those people who did not develop IA.
It is unclear where some of the inputs were obtained or derived. For example, patients who developed IA, entered the Markov model for the remaining months of the two-year time horizon. What does remaining months mean and how was this derived?

Based on data from the CSA cohorts Leiden and Rotterdam the mean time to develop IA was 5 months. So for a 2-year time horizon on average these patients spend 19 months in the Markov model (in total 24 months). For treated patients who developed IA a mean delay of 3 months was found in the TREAT EARLIER trial, so instead of 5 months in the CSA phase, these patients stayed 8 months in the CSA phase, and the remaining time (16 months) in the Markov model.

Since the time spend in the Markov models differed between patients who did develop IA and who did not develop IA we decided to use two separate Markov models for clarity. However, in theory it should not make a difference to use one Markov model with an additional health state of No IA. The outcomes would be the same because those patients could just transition within the no IA health state and to death.

6. Have the authors applied a half-cycle correction?

In the original manuscript we did not apply half-cycle correction but because it is common practice to do so, we decided to incorporate half-cycle correction. The results did not change considerably. We clarified that half-cycle correction was used in paragraph 2.4.1 (page 6): *"Half-cycle correction was applied to account for transitions occurring within cycles."*

7. Information is provided on the transition probabilities included in the model. Based on the Markov structure, people can transition to the dead state, but the transition matrices presented in Table S4 do not reflect this. What values are used for people transitioning to the dead state? Is there an increased risk of death for people who have IA compared to no IA.

We used background mortality based on the Dutch general population and did not increase the risk for people who had IA compared to no IA. Background mortality was age and sex specific based on CBS data[54]. At the averages ages of 44 and 48 the

background mortality is rather low and we did not expect it to be higher for patients at-risk of RA and who just developed RA compared to the general Dutch population.

The transition probabilities in table S4 were corrected for background mortality, see pages 33 and 34 of the manuscript.

8. Given that transition matrices (Table S4) are being used I would have expected there to be correlation between the parameters and hence the use of Cholesky decomposition in the probabilistic sensitivity analysis (PSA). Unless there is no correlation between the matrices (huge assumption), I am not convinced that the full uncertainty in the transition matrix is being captured.

For the transition probabilities we use a Dirichlet distribution for each row of the transition matrix. This ensures that the probabilities are jointly sampled and therefore captures the dependence between transition states within a row. For example, if the probability of transitioning from remission to low DAS increases, the probabilities of transitioning from remission to high DAS and/or remaining in remission decrease accordingly.

In Table S4, the transition probabilities are conditional on survival, because the death health state is fixed separately based on Dutch age and sex specific background mortality rates, therefore it is not included in the Dirichlet distribution.

9. Is the decision tree structure a reflection of the hybrid structure? If so, it might have been more appropriate to include a structure with the Markov nodes.

Yes, it is a hybrid structure and therefore we proposed a new figure 1 in which both parts of the model are shown in one figure. See the response to question 4, and figure 1 on page 8 of the manuscript.

Results

Authors reported deterministic and probabilistic sensitivity analyses results, which showed that all interventions were most costly and expected to yield more QALYs than the watchful waiting strategy.

10. From table 2, the QALYs yielded appear higher than expected given that the time horizon is only two years.

The yielded QALYs were 1.423 to 1.437, for autoantibody positive patients, meaning the average QALY per year is approximately 0.65, which does seem as expected given the utility values per health state (remission 0.7825, low DAS=0.7456, moderate/high DAS= 0.5997, and no IA= 0.7346). For autoantibody negative patients the yielded QALYs were slightly higher given their input values were slightly higher for the different DAS health states (remission 0.7892, low DAS=0.7102, moderate/high DAS= 0.6676, and no IA=

0.7346) and patients had a higher probability to move towards the remission health state and remain in the remission health state.

11. The authors stated that a 'full hierarchical' analysis had been undertaken. I take this to mean full incremental analysis. However, I am not convinced that these results are reported, and this is confirmed by the results reported in Table S9. The ICERs reported are pairwise ICERs for the comparisons between diagnostic imaging versus watchful waiting. Hence, there is no comparison between the diagnostic imaging strategies.

The ICERs are indeed based on a comparison of each strategy compared to watchful waiting. However, based on the results of table 2 (left side, full hierarchical analysis) the ICERs for all strategies compared to each other could be calculated (i.e., Conventional MRI vs Ultrasound (autoantibody negative patients): (€29,580-€29,286) / (1.451-1.449) = €147,000/QALY. Favoring ultrasound over conventional MRI. This can also be interpretated from the calculated NMBs and iNMBs.

12. It would have been useful to see results in terms of cost per diagnostic error avoided along with cost per life year and cost per QALY results.

In this context, we did not consider costs per diagnostic error avoided an appropriate outcome measure because individuals who did not develop IA but were treated (diagnostic error) did have a treatment effect in terms of improved QoL, lower medical consumption, and higher productivity.

13. **The authors stated that modified Dixon strategy was cost-effective with a probability of 49% at a WTP threshold of €50,000/QALY. However, it might be more appropriate to state that modified Dixon when compared to watchful waiting had a 0.49 probability of being cost-effective at a WTP threshold of €50,000/QALY.**

The results in figure 2 were not based on a comparison to watchful waiting alone but the 49% referred to the cost-effectiveness probability of modified Dixon compared to all other strategies combined. The results from figure 3 were compared to watchful waiting alone and the probability is 78% for autoantibody positive patients and 70% for autoantibody negative patients that the modified Dixon MRI strategy is cost-effective.

14. For people unfamiliar with presenting results in terms of NMB, the authors should state how these should be interpreted.

This should be explained in the method and we added it to paragraph 2.1.5. We added the following text on page 6: *"Other primary outcomes included the net monetary benefit (NMB) and incremental net monetary benefit (iNMB). The NMB was calculated by multiplying QALYs by the WTP threshold of €50,000 per QALY and subtracting the*

total costs of the strategy. The iNMB was defined as the difference in NMB between competing strategies."

15. Results from the PSA showed that a considerable number of iterations were in the southwest quadrant indicating that the intervention is less costly and less effective than watchful waiting; however, there is no discussion about that in the manuscript.

We incorporated this in the results and in the discussion. It is indeed uncertain whether treatment in the at-risk phase leads to QALY gains or losses, however the impact is rather small on the QoL and therefore we found gains or losses largely negligible. We did include a sentence in the results page 12: *"However, the 95% CIs were wide and very limited effects in terms of QALY gains were observed."*

However, it is important to reflect on the cost-effectiveness if QALY losses are not accepted against any cost-savings. See the proposed text in the discussion page 15: *"However, uncertainty is high (i.e., wide 95% CIs) and many cost-effectiveness iterations fell in the southwest quadrant (lower QALYs and cost savings). Nevertheless, if even very small QALY losses are considered unacceptable for at any level of costs savings, the probability for any of the intervention strategies to be cost-effective would still be approximately 50%."*

General comments

The manuscript contains lots of acronyms. Please can the authors consider not using non-standard acronyms. Examples, IA, CSA, UA, US, EAC

This makes sense, so we checked which acronyms could be avoided. We excluded UA, US, and EAC. We decided to keep IA (inflammatory arthritis) because this acronym was used many times and CSA because this acronym is relatively long (clinically suspect arthralgia).

VERSION 2 - REVIEW

Reviewer 1

Name Muller, Dirk

Affiliation University Hospital Cologne, Institute of Health Economics and Clinical Epidemiology

Date 29-May-2026

COI

The authors addressed most points of the first review, thereby clarifying the manuscript. Before the paper can be recommended for publications, some aspects remain to be revised:

• With regard to my first comment, there is still room for clarification. I pointed at the fact that in the TREAT EARLIER trial QoL via EQ-5D was assessed only as an explorative endpoint which should be mentioned. Given the limited evidence for an effect (in the TREAT EARLIER trial the frequency of the primary endpoint was similar between the groups and QALYs show limited effects with wide CIs), in the abstract the conclusion of your study should be more carefully (e.g., 'may cost-effective' instead of 'appears cost-effective'). In addition, the small and explorative treatment effect should be mentioned as a limitation in the discussion. Some statements in the discussion (e.g., 'a high probability to be cost-effective', 'robustness of results') should be revised or at least be more specified. According to the results, only for modified Dixon MRI vs watchful waiting the probability of being cost-effective is 70% (which not all readers or decision makers would consider as high). Avoiding an overoptimistic conclusion could prevent readers from misinterpretations.

• Response to comment 2: clarification in the manuscript would be useful.

• Abstract, 'Results' (last sentence): I suggest to say: 'Deterministic sensitivity analysis confirmed robustness but treatment effects and treatment disutilities were sensitive parameters'.

• Page 89, line20-22: the explanation could be more precise (all transitions occur within cycles).

• Section 2.4.4.: the heading of the section addresses economic aspects with some sentences targeting effects (e.g., line 13). These sentences should be deleted or placed in another section (what is meant with '..but additional subgroup analyses were performed'?).

**Reviewer**	**2**
**Name**	**Auguste, Peter**
**Affiliation**	**University of Warwick Warwick Medical School**
**Date**	**11-Jun-2026**
**COI**	

Many thanks to the authors for carefully considering and responding to the peer reviewers' comments. Additionally, for making the necessary amendments to the manuscript.

There are no further queries, but I wonder if a limitation of this work is the ?assumption that people who had a false positive result and treated experienced a treatment effect. Could this be potentially be an over-estimate of the treatment effect? It would have been interesting to undertake a scenario analysis, if possible, which excludes this treatment effect for these people.

VERSION 2 - AUTHOR RESPONSE

Reviewer: 1
Dr. Dirk Muller, University Hospital Cologne
Comments to the Author:
The authors addressed most points of the first review, thereby clarifying the manuscript. Before the paper can be recommended for publications, some aspects remain to be revised:

- **With regard to my first comment, there is still room for clarification. I pointed at the fact that in the TREAT EARLIER trial QoL via EQ-5D was assessed only as an explorative endpoint which should be mentioned. Given the limited evidence for an effect (in the TREAT EARLIER trial the frequency of the primary endpoint was similar between the groups and QALYs show limited effects with wide CIs), in the abstract the conclusion of your study should be more carefully (e.g., 'may cost-effective' instead of 'appears cost-effective'). In addition, the small and explorative treatment effect should be mentioned as a limitation in the discussion. Some statements in the discussion (e.g., 'a high probability to be cost-effective', 'robustness of results') should be revised or at least be more specified. According to the results, only for modified Dixon MRI vs watchful waiting the probability of being cost-effective is 70% (which not all readers or decision makers would consider as high). Avoiding an overoptimistic conclusion could prevent readers from misinterpretations.**

We adjusted our conclusion in the abstract and we revised some of the statements in the discussion to be more cautious. We acknowledge that the treatment effect on QoL measured with the EQ-5D is limited, however the main driver for cost-effectiveness is cost-savings due to productivity gains instead of improved HRQoL. We reflect on this in the discussion. We also agree that in the TREAT EARLIER trial no difference in the primary endpoint was found between the intervention and control group. However, given that the trial did show improved MRI-detected subclinical inflammation scores, improved physical functioning, and less pain for the intervention group compared to the control group, we do expect that the minor improvement in utility score (i.e. 0.0202) is clinically plausible. We do acknowledge uncertainty and wide Cis by showing the CE-planes of the PSA and the outcomes of the one-way sensitivity analyses. Furthermore, we did include a scenario analysis in which we assumed no treatment effect related to QoL, medical consumption, and productivity because of the wide Cis. We do state that the intervention strategies are not cost-effective compared to watchful waiting if this scenario were to be true.

Abstract: Conclusions: Imaging-guided early treatment for arthralgia at-risk for RA *may be* cost-effective, particularly for autoantibody-positive patients, irrespective of imaging modality. Modified Dixon MRI seems most promising and requires further validation in CSA.

Discussion: *Our analyses, conducted from a societal perspective, demonstrated that diagnosing subclinical inflammation and initiating treatment with a single intramuscular*

glucocorticoid injection (120mg) and a one-year course of oral MTX for individuals with positive subclinical inflammation scores, had a 50% to 78% probability to be cost-effective compared to watchful waiting, regardless of imaging modality used.

The OWSA confirmed these results, with most parameters resulting in positive NMBs across the tested input ranges. The only exceptions were the treatment effect on productivity and quality of life, which caused the diagnostic strategies not to be cost-effective at the most conservative input values.

Our analysis indicates that early intervention in CSA patients, guided by imaging, *may be* cost-effective.

In conclusion, imaging-guided detection of high-risk CSA patients combined with treatment with a single intramuscular glucocorticoid injection (120mg) and a one-year course of oral MTX, may be cost-effective from the societal perspective

- **Response to comment 2: clarification in the manuscript would be useful.**

We added some additional clarification in the manuscript, in paragraph 2.4.1. *Firstly, the 2-year results from the TREAT EARLIER trial showed that treatment delayed the time to IA development by 3 months, as the mean time to IA development in the intervention group was 13 months and in the placebo group 10 months over the two-year time period[21]. Hence, in the base-case of the model CSA patients who were treated developed IA on average after 8 months instead of 5 months because in the CSA cohorts the average time to IA development was 5 months.*

- **Abstract, 'Results' (last sentence): I suggest to say: 'Deterministic sensitivity analysis confirmed robustness but treatment effects and treatment disutilities were sensitive parameters'.**

We changed this sentence in the abstract: "*Deterministic sensitivity analysis showed the results were robust to most changes in parameters however, the results were sensitive to extreme changes in parameters related to treatment effects on QoL and productivity and disutilities due to treatment-related side-effects*". The results were sensitive to changes in those parameters.

- **Page 89, line20-22: the explanation could be more precise (all transitions occur within cycles).**

We changed the sentence to: "*Half-cycle correction was applied to assume that, on average, transitions occur halfway through each cycle*".

• Section 2.4.4.: the heading of the section addresses economic aspects with some sentences targeting effects (e.g., line 13). These sentences should be deleted or placed in another section (what is meant with '..but additional subgroup analyses were performed'?).

We have added the parts of the treatment effect for QALYs, productivity, and medical consumption to treatment effects instead of mentioning them in the separate sections. We added this to paragraph 2.4.1: *Thirdly, we modelled a positive treatment effect on EQ-5D utilities, medical consumption, and productivity for all patients with subclinical inflammation on the imaging tests who were given treatment. This effect was obtained from the TREAT EARLIER trial, which found these effects in treated CSA patients, irrespective of IA development[23]*. *The effect of treating CSA patients with subclinical inflammation was an improvement in utility of 0.0202 over a period of two years for autoantibody-positive patients and 0.0200 for autoantibody-negative patients, based on a subgroup analysis (i.e., autoantibody status) of the TREAT EARLIER trial [23]. This treatment effect was the mean effect across all treated CSA patients in this trial irrespective of development of IA. For treated autoantibody-positive patients, the medical consumption costs were found to be 89% of the costs for untreated patients, and productivity costs were found to be 75%. For treated autoantibody-negative patients, these percentages were 79% and 95%, respectively. As the TREAT EARLIER trial did not assess treatment effects on informal care, no effect on costs of informal care was applied in the health economic model.*

With 'additional subgroup analyses' we referred to subgroup analysis of costs in the TREAT EARLIER trial by autoantibody status (ACPA and/or RF positive and ACPA and RF negative).

Reviewer: 2
Mr. Peter Auguste, University of Warwick Warwick Medical School
Comments to the Author:
Many thanks to the authors for carefully considering and responding to the peer reviewers' comments. Additionally, for making the necessary amendments to the manuscript.

There are no further queries, but I wonder if a limitation of this work is the ?assumption that people who had a false positive result and treated experienced a treatment effect. Could this be potentially be an over-estimate of the treatment effect? It would have been interesting to undertake a scenario analysis, if possible, which excludes this treatment effect for these people.

We had also considered exploring a scenario in which a treatment effect would only be assigned to individuals who actually developed IA. However, we did not include this because the treatment effect in the TREAT EARLIER trial was estimated on the whole trial population,

including the individuals not developing IA. If we just assume a treatment effect for individuals who developed IA (44% in the autoantibody positive group and 9% in the autoantibody negative group) we would underestimate the treatment effect. We already included a more conservative scenario in which we do not assume any treatment effect on QoL, medical consumption and productivity because of uncertainty, wide CIs, and a large proportion of individuals not developing IA (i.e., scenario 3). Nevertheless, we followed your suggestion by adding an additional (fourth) scenario in which treatment effect on EQ-5D utilities, medical consumption, and productivity were only assigned to individuals developing IA. In this scenario, we found that the intervention is still cost-effective in autoantibody positive patients but not in autoantibody negative patients, which makes sense considering the low percentage of patients developing IA. Below you find the text included in the manuscript:

Paragraph 2.7: scenario analyses

Scenario 4: Only treatment effects for individuals developing IA: additional to scenario 3 we also investigated the cost-effectiveness when applying the trial-wide treatment effects on quality of life, medical consumption, and productivity to individuals who developed IA only. This is highly conservative, since the trial-wide effect was estimated on all patients and not only those developing IA. We do not know the true treatment effect in this subgroup.

Paragraph 3.4

The tables and figures in supplemental file 9 showed that scenario 2, a relative delay in time-to-IA-diagnosis, had no effect on the outcomes of the cost-utility analysis. In contrast, scenario 3 notably impacted cost-effectiveness results: when no improvement in health-related quality of life and no reduction in productivity loss and healthcare utilization was assumed, none of the intervention strategies were cost-effective. *However, scenario 4 showed that when the treatment effect on quality of life, medical consumption, and productivity were only assumed for individuals developing IA, all intervention strategies were cost-effective compared to watchful waiting for autoantibody positive patients but none were cost-effective for autoantibody negative patients because only 9% of these individuals developed IA.*

Discussion

Further validation was provided by the four scenario analyses, which generally supported our results. However, when a one-time disutility due to treatment-related side-effects was introduced, cost-effectiveness depended on both the proportion of patients affected and the magnitude of disutility, highlighting the importance of investigating this further. When only modelling the effect of the diagnostic interventions in terms of the delay in time-to-IA-diagnosis and simultaneously leaving out treatment effects on quality-of-life, productivity, and medical consumption, none of the intervention strategies remained cost-effective. *Additionally, when these treatment effect were only assigned to individuals who developed*

IA, the intervention strategies were only cost-effective compared to watchful waiting for autoantibody positive patients. While *these two scenarios* probably underestimate the treatment effect, given the TREAT EARLIER trial demonstrated positive effects *on quality of life, medical consumption and productivity*, caution is needed, as these analyses were done in the total trial population and recent data has shown that the total CSA population is heterogeneous in pathophysiology and risk of RA [59–61]. This appears to blur treatment effects for certain subgroups. ACPA-negative CSA patients at increased risk for RA, as determined by the presence of extensive subclinical joint inflammation on MRI, were found to have the highest treatment effectiveness, with benefits that reduced both RA progression and disease burden [59,62]. Patients who were ACPA-positive and at increased risk for RA had a temporary benefit for 2 years, but not for 5-years, while patients who were retrospectively at low risk for RA had no benefit at all[59,62]. This could mean that in our analysis the overall treatment effect may have been diluted by including a substantial proportion of autoantibody-negative low risk individuals (i.e., patients with low subclinical inflammation scores). Hence, targeting high-risk subgroups may improve clinical and economic benefits of early intervention. Importantly, MRI was important for risk prediction; the number of locations of subclinical inflammation, and presence of the metacarpophalangeal-extensor tenosynovitis [59,63] were crucial predictors and these can only be accurately detected using MRI and not through US.

VERSION 3 - REVIEW

**Reviewer**	**2**
**Name**	**Auguste, Peter**
**Affiliation**	**University of Warwick Warwick Medical School**
**Date**	**10-Aug-2026**
**COI**	

Many thanks for considering and carefully responding to the reviewers' comments and, undertaking additional scenario analysis. The comments have all been addressed and changes made to the manuscript are all appropriate.

I have no further comments.
