# Supplementary material for: Imaging-guided treatment strategy compared to standard care in at-risk individuals for rheumatoid arthritis: a cost-utility analysis
Source: BMJ Open. 2026 Oct 21;16(10):e118495. doi: 10.1136/bmjopen-2026-118495 (PMC13691768; doi:10.1136/bmjopen-2026-118495)
Supplement: Supplementary file 2 [file bmjopen-16-10-s001.docx]

**Supplemental file 1: CHEERS checklist**

Table S1. CHEERS 2022 checklist

| **Topic** | **No.** | **Item** | **Location where item is reported** |
| --- | --- | --- | --- |
| **Title** |  |  |  |
|  | 1 | Identify the study as an economic evaluation and specify the interventions being compared. | Title, Page 1 |
| **Abstract** |  |  |  |
|  | 2 | Provide a structured summary that highlights context, key methods, results, and alternative analyses. | Abstract, Page 1 |
| **Introduction** |  |  |  |
| **Background and objectives** | 3 | Give the context for the study, the study question, and its practical relevance for decision making in policy or practice. | Introduction, Page 2 |
| **Methods** |  |  |  |
| **Health economic analysis plan** | 4 | Indicate whether a health economic analysis plan was developed and where available. | Not reported |
| **Study population** | 5 | Describe characteristics of the study population (such as age range, demographics, socioeconomic, or clinical characteristics). | Methods, Paragraph 2.2 |
| **Setting and location** | 6 | Provide relevant contextual information that may influence findings. | Methods, Paragraph 2.2.1 |
| **Comparators** | 7 | Describe the interventions or strategies being compared and why chosen. | Methods, Paragraph 2.2.2 & 2.2.3 |
| **Perspective** | 8 | State the perspective(s) adopted by the study and why chosen. | Methods, Paragraph 2.1.4 |
| **Time horizon** | 9 | State the time horizon for the study and why appropriate. | Methods, Paragraph 2.1.4 |
| **Discount rate** | 10 | Report the discount rate(s) and reason chosen. | Methods, Paragraph 2.1.4 |
| **Selection of outcomes** | 11 | Describe what outcomes were used as the measure(s) of benefit(s) and harm(s). | Methods, Paragraph 2.1.4 & 2.1.5 |
| **Measurement of outcomes** | 12 | Describe how outcomes used to capture benefit(s) and harm(s) were measured. | Methods, Paragraph 2.4 |
| **Valuation of outcomes** | 13 | Describe the population and methods used to measure and value outcomes. | Methods, Paragraph 2.4 |
| **Measurement and valuation of resources and costs** | 14 | Describe how costs were valued. | Methods, Paragraph 2.1.4 |
| **Currency, price date, and conversion** | 15 | Report the dates of the estimated resource quantities and unit costs, plus the currency and year of conversion. | Methods, Paragraph 2.1.4 |
| **Rationale and description of model** | 16 | If modelling is used, describe in detail and why used. Report if the model is publicly available and where it can be accessed. | Methods, Paragraph 2.2 |
| **Analytics and assumptions** | 17 | Describe any methods for analysing or statistically transforming data, any extrapolation methods, and approaches for validating any model used. | Methods , Paragraph 2.4 and 2.5 |
| **Characterising heterogeneity** | 18 | Describe any methods used for estimating how the results of the study vary for subgroups. | Methods, Paragraph 2.2 |
| **Characterising distributional effects** | 19 | Describe how impacts are distributed across different individuals or adjustments made to reflect priority populations. | Not reported |
| **Characterising uncertainty** | 20 | Describe methods to characterise any sources of uncertainty in the analysis. | Methods, Paragraph 2.6 & 2.7 |
| **Approach to engagement with patients and others affected by the study** | 21 | Describe any approaches to engage patients or service recipients, the general public, communities, or stakeholders (such as clinicians or payers) in the design of the study. | Methods, Paragraph 2.5 |
| **Results** |  |  |  |
| **Study parameters** | 22 | Report all analytic inputs (such as values, ranges, references) including uncertainty or distributional assumptions. | Results, appendix 2 |
| **Summary of main results** | 23 | Report the mean values for the main categories of costs and outcomes of interest and summarise them in the most appropriate overall measure. | Results, Paragraph 3.1 |
| **Effect of uncertainty** | 24 | Describe how uncertainty about analytic judgments, inputs, or projections affect findings. Report the effect of choice of discount rate and time horizon, if applicable. | Results, Paragraph 3.2 & 3.4 |
| **Effect of engagement with patients and others affected by the study** | 25 | Report on any difference patient/service recipient, general public, community, or stakeholder involvement made to the approach or findings of the study | Not reported |
| **Discussion** |  |  |  |
| **Study findings, limitations, generalisability, and current knowledge** | 26 | Report key findings, limitations, ethical or equity considerations not captured, and how these could affect patients, policy, or practice. | Discussion |
| **Other relevant information** |  |  |  |
| **Source of funding** | 27 | Describe how the study was funded and any role of the funder in the identification, design, conduct, and reporting of the analysis | End of manuscript |
| **Conflicts of interest** | 28 | Report authors conflicts of interest according to journal or International Committee of Medical Journal Editors requirements. | End of manuscript |

*From:* Husereau D, Drummond M, Augustovski F, et al. Consolidated Health Economic Evaluation Reporting Standards 2022 (CHEERS 2022) Explanation and Elaboration: A Report of the ISPOR CHEERS II Good Practices Task Force. Value Health 2022;25. <doi:10.1016/j.jval.2021.10.008>[27]

**Supplemental file 2: Cohorts and TREAT EARLIER trial**

The Leiden CSA cohort[45] and the multicenter Rotterdam CSA cohort[46] are two independent cohorts that are designed similarly. Patients were included from 2012 and 2017 onwards, respectively. Eligible patients had to have arthralgia of the small joints (symptom duration <1 year) that was considered a potential risk for progression to rheumatoid arthritis (RA) by the rheumatologist. If another explanation for the complaints was more likely than imminent RA, patients were not included. Patients were followed for two years or until they developed clinically apparent inflammatory arthritis (IA), confirmed with joint swelling at physical examination by the rheumatologist. Visits were scheduled at time of inclusion, after 4 months, 12 months, and 24 months, or when patients had increased symptoms. During follow-up, CSA-patients were not treated with DMARDs (also no glucocorticoids). Since patients are still being included in both cohorts at the time of this analysis, we only included CSA-patients who had a minimum follow-up time of 6 months for our analyses.

For the period after IA development, input parameters were obtained from the early arthritis clinic cohort from Leiden[48,70]. This cohort included patients with early arthritis at physical examination by the rheumatologist and a symptom duration of less than two years from 1993 onwards. For the current study, we selected patients with IA, defined as either RA (fulfilling the 1987 and/or 2010 criteria), or undifferentiated arthritis (arthritis, but not fulfilling 1987 and/or 2010 criteria).

The TREAT EARLIER trial was a randomized, double-blind, placebo-controlled trial conducted between April 2015 and September 2019 at the Leiden University Medical Center, the Netherlands[21,39]. Eligible patients had to have recent onset of arthralgia in the small joints (less than 1 year of complaints) and be suspicious for developing RA according to the rheumatologist. Besides arthralgia, patients also had to show subclinical inflammation on the conventional MRI scan. This was defined as having at least one joint tissue (synovitis, tenosynovitis, osteitis) with inflammation on MRI, scored by two independent blinded readers, present in <5% of the age-matched, symptom-free healthy population at the same location[39]. After inclusion in the trial (n=236), patients were evenly randomized to one dose of intramuscular glucocorticoids and one year treatment with methotrexate or placebo, followed by a second year without treatment. The primary endpoint of the trial was development of persistent clinically apparent IA. This was defined as joint swelling confirmed by two rheumatologists, which needed to be confirmed after two weeks to determine persistent inflammatory arthritis. When the primary endpoint was reached, patients exited the study.

Figure S1 shows an overview of which data was used in each part of the model.

**Figure S1. Data sources used for each part of the model.**

CSA population

Watchful waiting

CSA phase (inputs based on CSA cohort Leiden and Rotterdam)

No treatment until swollen joints

IA (inputs based on EAC cohort) -	Start Treatment with MTX

No IA (inputs based on CSA cohort Leiden and Rotterdam)

MRI, modified Dixon MRI, and US

Positive imaging (MRI based on CSA cohort Leiden, modified Dixon the same as MRI29, and US based on comparison to MRI39)

CSA phase (inputs based on CSA cohort Leiden and Rotterdam combined with treatment effect found in the TREAT EARLIER trial)

Treatment

IA (10%-point more patients start in a lower DAS based on the TREAT EARLIER trial. The inputs were based on EAC cohort) - Contininue treatment with MTX

No IA (inputs based on CSA cohort Leiden + CSA cohort Rotterdam, combined with the treatment effect of the TREAT EARLIER trial) - Stop treatment after one year

Negative imaging (MRI based on CSA cohort Leiden, modified Dixon the same as MRI29, and US based on comparison to MRI39)

CSA phase (inputs based on CSA cohort Leiden and Rotterdam)

No treatment until swollen joints

IA (inputs based on EAC cohort) -	Start treatment with MTX

No IA (inputs based on CSA cohort Leiden and Rotterdam)

**CSA= clinically suspect arthralgia, MRI= magnetic resonance imaging, US= ultrasound, IA= inflammatory arthritis (i.e., rheumatoid arthritis and undifferentiated arthritis), EAC= early arthritis clinic, and MTX= methotrexate.*

**Supplemental file 3: Input parameters**

Tables S2a and S2b provide the sensitivity and specificity of MRI and ultrasound for both autoantibody-positive and -negative individuals, table S3 provides the health states in which individuals start the Markov model, table S4 provides the transition probabilities, table S5 provides the quality-of-life estimates, table S6 provides the one-time intervention costs, and table S7 provides the medical consumption costs, productivity costs, and informal care costs.

**Table S2a. Sensitivity and specificity imaging modalities**

| Variable name | Parameters | Mean value (SE) | Distribution | Distribution parameter | Source |
| --- | --- | --- | --- | --- | --- |
| Autoantibody-positive patients | | | | |  |
| P_ww_IA | Probability of developing IA | 0.44 (0.03) | Beta | α =93, β =119 | CSA cohort Leiden & Rotterdam |
| Conventional MRI | |  |  |  |  |
| MRI_true_pos | Sensitivity MRI | 0.83 (0.05) | Beta | α =39, β =8 | Leiden CSA cohort |
| MRI_true_neg | Specificity MRI | 0.68 (0.05) | Beta | α =50, β =24 | Leiden CSA cohort |
| Modified Dixon MRI | | | | | |
| MD_MRI_true_pos | Sensitivity MD MRI | 0.83 (0.16) | Beta | α =4, β =1 | *Same as sensitivity conventional MRI [38] |
| MD_MRI_true_neg | Specificity MD MRI | 0.68 (0.13) | Beta | α =8, β =4 | *Same as specificity conventional MRI [38] |
| Ultrasound | |  |  |  |  |
| US_ohrndorf_sens | Sensitivity US compared to MRI | 0.47 (0.11) | Beta | α =9, β =10 | **Ohrndorf et al[54] |
| US_ohrndorf_spec | Specificity US compared to MRI | 0.90 (0.08) | Beta | α =12, β =1 | **Ohrndorf et al[54] |
| US_true_pos | Base-case: Sensitivity US | 0.41 | - | - | ***Calculated |
| US_true_neg | Base-case: Specificity US | 0.78 | - | - | ****Calculated |
| Autoantibody-negative patients | | | | |  |
| P_ww_IA | Probability of developing IA | 0.09 (0.01) | Beta | α =61, β =615 | CSA cohort Leiden & Rotterdam |
| Conventional MRI | |  |  |  |  |
| MRI_true_pos | Sensitivity MRI | 0.65 (0.07) | Beta | α =29, β =15 | Leiden CSA cohort |
| MRI_true_neg | Specificity MRI | 0.70 (0.02) | Beta | α =303, β =130 | Leiden CSA cohort |
| Modified Dixon MRI | | | | | |
| MD_MRI_true_pos | Sensitivity MD MRI | 0.65 (0.12) | Beta | α =10, β =5 | Sensitivity MRI |
| MD_MRI_true_neg | Specificity MD MRI | 0.70 (0.14) | Beta | α =7, β =3 | Specificity MRI |
| Ultrasound | | |  |  |  |
| US_ohrndorf_sens | Sensitivity US compared to MRI | 0.47 (0.11) | Beta | α =9, β =10 | **Ohrndorf et al[54] |
| US_ohrndorf_spec | Specificity US compared to MRI | 0.90 (0.08) | Beta | α =12, β =1 | **Ohrndorf et al[54] |
| US_true_pos | Base-case: Sensitivity US | 0.34 | - | - | ***Calculated |
| US_true_neg | Base-case: Specificity US | 0.79 | - | - | ****Calculated |

**Although we assumed the same sensitivity and specificity for MD MRI and conventional MRI based on Boeren et al[38], the SEs of the sensitivity and specificity of the MD MRI were unknown. We assumed more uncertainty around these estimates for the modified Dixon MRI compared to the conventional MRI. Therefore, we used a SE of 20% of the mean value.*

***Summed inflammation score of CSA patients (n=40) based on the same data used in the study of Ohrndorf et al, see also supplemental table S2b. In the CSA group, in total 19 patients had a positive MRI and 11 patients had a positive US. The sensitivity was calculated to be 47% (9/19*100%) and the specificity 90% (19/21*100%)*

*****US_true_pos **=** *MRI_true_positive * US_ohrndorf_sens + MRI_false_neg * (1- US_ohrndorf_spec)*

******US_true_neg **=** *MRI_true_negative * US_ohrndorf_spec + MRI_false_pos * (1- US_ohrndorf_sens)*

**Table S2b. Sensitivity and specificity ultrasound compared to MRI in CSA patients, using original data from Ohrndorf et al** [54,65]

|  |  | MRI | |  |
| --- | --- | --- | --- | --- |
|  |  | **negative** | **positive** | **Total** |
| US | **negative** | 19 | 10 | **29** |
|  | **positive** | 2 | 9 | **11** |
|  | **Total** | **21** | **19** |  |
| Sensitivity | 0.47 (0.27-0.68) | | |  |
| Specificity | 0.90 (0.71-0.97) | | |  |

**Accuracy was assessed in CSA patients. A positive ultrasound or MRI was based on joints from hands and wrists.*

**Table S3. Initial health states and treatment effect on the initial health states.**

| Variable name | Parameter | Mean value | Distribution | Distribution parameter | Source |
| --- | --- | --- | --- | --- | --- |
| Initial health states untreated autoantibody-positive CSA patients | | | | |  |
| v_init_ww_IA | Remission | 0.05 | Dirichlet | Αlpha_1=29 | EAC cohort Leiden |
| v_init_ww_IA | Low DAS | 0.13 | Dirichlet | Αlpha_2=69 | EAC cohort Leiden |
| v_init_ww_IA | Moderate/high DAS | 0.82 | Dirichlet | Αlpha_3=437 | EAC cohort Leiden |
| Initial health states untreated autoantibody-negative CSA patients | | | | | |
| v_init_ww_IA | Remission | 0.10 | Dirichlet | Αlpha_1=76 | EAC cohort Leiden |
| v_init_ww_IA | Low DAS | 0.24 | Dirichlet | Αlpha_2=182 | EAC cohort Leiden |
| v_init_ww_IA | Moderate/high DAS | 0.66 | Dirichlet | Αlpha_3=512 | EAC cohort Leiden |
| Treatment effect rightfully treated CSA patients | | | | | |
| v_init_MRI_IA* | Remission | +0 |  |  | TREAT EARLIER |
| v_init_MRI_IA* | Low DAS | +10%-point | Triangular | Min=0, max=0.50, mode = 0.10 | TREAT EARLIER |
| v_init_MRI_IA* | Moderate/ high DAS | - 10%-point | Triangular | Min=-0.50, max=0, mode = -0.10 | TREAT EARLIER |

**This is the same for modified Dixon MRI (v_init_MD_MRI_IA), and ultrasound (v_init_US_IA) strategies.*

**Table S4. Transition probabilities.**

| Variable name | Transition probabilities | Mean value | Distribution | Distribution parameter | Source |
| --- | --- | --- | --- | --- | --- |
| Autoantibody-positive patients | | | | |  |
|  | **Remission to** |  |  |  |  |
| P_low_low | Remission | (1-background mortality) * 0.69 | Dirichlet | Αlpha_1=179 | EAC cohort Leiden |
| P_low_mod | Low DAS | (1-background mortality) * 0.10 | Dirichlet | Αlpha_2=32 | EAC cohort Leiden |
| P_low_high | Moderate/high DAS | (1-background mortality) * 0.21 | Dirichlet | Αlpha_3=55 | EAC cohort Leiden |
| P_low_death | Death | Background mortality |  |  | CBS [55] |
|  | **Low DAS to** |  |  |  |  |
| P_mod_low | Remission | (1-background mortality) * 0.44 | Dirichlet | Αlpha_1=77 | EAC cohort Leiden |
| P_mod_mod | Low DAS | (1-background mortality) * 0.24 | Dirichlet | Αlpha_2=42 | EAC cohort Leiden |
| P_mod_high | Moderate/high DAS | (1-background mortality) * 0.32 | Dirichlet | Αlpha_3= 55 | EAC cohort Leiden |
| P_mod_death | Death | Background mortality |  |  | CBS [55] |
|  | **Moderate/high DAS to** |  |  |  |  |
| P_high_low | Remission | (1-background mortality) * 0.31 | Dirichlet | Αlpha_1=205 | EAC cohort Leiden |
| P_high_mod | Low DAS | (1-background mortality) * 0.19 | Dirichlet | Αlpha_2=122 | EAC cohort Leiden |
| P_high_high | Moderate/high DAS | (1-background mortality) * 0.50 | Dirichlet | Αlpha_3=314 | EAC cohort Leiden |
| P_high_death | Death | Background mortality |  |  | CBS [55] |
| Autoantibody-negative patients | | | | |  |
|  | **Remission to** |  |  |  |  |
| P_low_low | Remission | (1-background mortality) * 0.74 | Dirichlet | Αlpha_1=212 | EAC cohort Leiden |
| P_low_mod | Low DAS | (1-background mortality) * 0.08 | Dirichlet | Αlpha_2=24 | EAC cohort Leiden |
| P_low_high | Moderate/high DAS | (1-background mortality) * 0.18 | Dirichlet | Αlpha_3=46 | EAC cohort Leiden |
| P_low_death | Death | Background mortality |  |  | CBS [55] |
|  | **Low DAS to** |  |  |  |  |
| P_mod_low | Remission | (1-background mortality) * 0.44 | Dirichlet | Αlpha_1=107 | EAC cohort Leiden |
| P_mod_mod | Low DAS | (1-background mortality) * 0.29 | Dirichlet | Αlpha_2=73 | EAC cohort Leiden |
| P_mod_high | Moderate/high DAS | (1-background mortality) * 0.27 | Dirichlet | Αlpha_3= 49 | EAC cohort Leiden |
| P_mod_death | Death | Background mortality |  |  | CBS [55] |
|  | **Moderate/high DAS to** |  |  |  |  |
| P_high_low | Remission | (1-background mortality) * 0.32 | Dirichlet | Αlpha_1=210 | EAC cohort Leiden |
| P_high_mod | Low DAS | (1-background mortality) * 0.19 | Dirichlet | Αlpha_2=123 | EAC cohort Leiden |
| P_high_high | Moderate/high DAS | (1-background mortality) * 0.49 | Dirichlet | Αlpha_3=312 | EAC cohort Leiden |
| P_high_death | Death | Background mortality |  |  | CBS [55] |

**Table S5. Health-related quality of life in the CSA phase and after the development of IA.**

| Variable name | Parameters | Mean value (SE) | Distribution | Distribution parameter | Source |
| --- | --- | --- | --- | --- | --- |
| CSA phase |  |  |  |  |  |
| u_ww_CSA_IA | 5 months in the CSA phase | 0.287 (0.03) | Beta | α =71, β =176 | CSA cohort Leiden & Rotterdam published in Bour et al[49] |
| u_MRI_CSA_IA* | 6.7 months in the CSA phase (relative delay) | 0.389 (0.06) | Beta | α =27, β =42 | CSA cohort Leiden & Rotterdam published in Bour et al[49] |
| u_MRI_CSA_IA* | 8 months in the CSA phase (absolute delay) | 0.468 (0.07) | Beta | α =23, β =26 | CSA cohort Leiden & Rotterdam published in Bour et al[49] |
| Per health state (per year) – autoantibody-positive patients | | | | |  |
| u_ww_low_DAS | Remission | 0.7825 (0.08) | Beta | α =21 , β =6 | EAC cohort Leiden published in Bour et al[49] |
| u_ww_mod_DAS | Low DAS | 0.7456 (0.05) | Beta | α =50, β =17 | EAC cohort Leiden published in Bour et al[49] |
| u_ww_high_DAS | Moderate/ high DAS | 0.5997 (0.05) | Beta | α =55, β =37 | EAC cohort Leiden published in Bour et al[49] |
| u_ww_no_IA | No IA | 0.7346 (0.01) | Beta | α =952 , β =344 | CSA cohort Leiden & Rotterdam published in Bour et al[49] |
| Per health state (per year) – autoantibody-negative patients | | | | |  |
| u_ww_low_DAS | Remission | 0.7892 (0.04) | Beta | α =69, β =18 | EAC cohort Leiden published in Bour et al[49] |
| u_ww_mod_DAS | Low DAS | 0.7102 (0.06) | Beta | α =38, β =15 | EAC cohort Leiden published in Bour et al[49] |
| u_ww_high_DAS | Moderate/ high DAS | 0.6676 (0.05) | Beta | α =52 , β =26 | EAC cohort Leiden published in Bour et al[49] |
| u_ww_no_IA | No IA | 0.7346 (0.01) | Beta | α =952 , β =344 | CSA cohort Leiden & Rotterdam published in Bour et al[49] |
| Treatment effect | | | | | |
| u_treatment_  effect_lower | Treated autoantibody- positive CSA patients over two years | 0.0202 | Triangular | Min=-0.1143, max=0.1528, mean = 0.0202) | TREAT EARLIER trial |
| u_treatment_  effect_lower | Treated autoantibody- negative CSA patients over two years | 0.0200 | Triangular | Min=-0.0683, max=0.1064, mean = 0.0200) | TREAT EARLIER trial |

**This is the same for modified Dixon MRI (U_MD_MRI_CSA_IA), and ultrasound (U_US_CSA_IA) strategies*

**Table S6. One-time intervention costs.**

| Variable name | Parameters | Mean value | Distribution | Distribution parameter | Source |
| --- | --- | --- | --- | --- | --- |
| Costs diagnostics |  |  |  |  |  |
| MRI_costs | MRI for two hands | €537 | Gamma | Shape= 100, scale= 5 | Dutch costing manual |
| MD_MRI_costs | modified Dixon MRI | €110 | Gamma | Shape= 25, scale= 4 | Expert opinion |
| US_costs | Ultrasound | €103 | Gamma | Shape= 100, scale= 1 | Dutch costing manual |
| Costs treatment in intervention strategies | | | | | |
| Treatment_costs | Treatment with MTX and a single intramuscular glucocorticoid injection | €68 | Gamma | Shape= 100, scale= 0.68 | TREAT EARLIER trial |

**Table S7. Medical consumption costs, productivity costs, and informal care costs.**

| Variable name | Parameters | Mean value (SE) | Distribution | Distribution parameter | Source |
| --- | --- | --- | --- | --- | --- |
| Medical consumption costs | | | | |  |
| CSA phase |  |  |  |  |  |
| hc_ww_CSA_IA | 5 months in the CSA phase | €1267 (127) | Gamma | Shape= 100, scale= 13 | CSA cohorts |
| hc_MRI_CSA_IA* | 6.7 months in the CSA phase (relative treatment effect) | €1723 (172) | Gamma | Shape= 100, scale=17 | CSA cohorts |
| hc_MRI_CSA_IA* | 8 months in the CSA phase (absolute treatment effect) | €2086 (208) | Gamma | Shape= 100, scale= 21 | CSA cohorts |
| Per health state (3 months) – Autoantibody-positive | | | | |  |
| hc_ww_low_DAS | Remission | €406 (380) | Gamma | Shape= 1, scale= 356 | EAC cohort |
| hc_ww_mod_DAS | Low DAS | €658 (314) | Gamma | Shape= 3, scale= 173 | EAC cohort |
| hc_ww_high_DAS | Moderate/ high DAS | €1278 (305) | Gamma | Shape= 18, scale=73 | EAC cohort |
| hc_ww_no_IA | No IA | €543 (75) | Gamma | Shape= 52, scale= 10 | CSA cohorts |
| Per health state (3 months) – Autoantibody-negative | | | | |  |
| hc_ww_low_DAS | Remission | €559 (167) | Gamma | Shape= 11, scale= 50 | EAC cohort |
| hc_ww_mod_DAS | Low DAS | €366 (239) | Gamma | Shape= 2, scale= 183 | EAC cohort |
| hc_ww_high_DAS | Moderate/ high DAS | €733 (292) | Gamma | Shape= 6, scale=117 | EAC cohort |
| hc_ww_no_IA | No IA | €543 (75) | Gamma | Shape= 52, scale= 10 | CSA cohorts |
| Treatment effect |  |  |  |  |  |
| hc_treatment_effect_ lower | Treated autoantibody-positive CSA patients | 89.0% | Triangular | Min= 34%, max=151%, mean = 89.0%) | TREAT EARLIER trial |
| hc_treatment_effect_ lower | Treated autoantibody-negative CSA patients | 78.8% | Triangular | Min= 58%, max=110%, mean = 78.8%) | TREAT EARLIER trial |
| Productivity costs |  |  |  |  |  |
| CSA phase |  |  |  |  |  |
| pc_ww_CSA_IA | 5 months in the CSA phase | €9591 (959) | Gamma | Shape= 100, scale= 96 | CSA cohorts |
| pc_MRI_CSA_IA* | 6.7 months in the CSA phase (relative treatment effect) | €12366 (1237) | Gamma | Shape=100, scale= 124 | CSA cohorts |
| pc_MRI_CSA_IA* | 8 months in the CSA phase (absolute treatment effect) | €14353 (1435) | Gamma | Shape= 100, scale= 144 | CSA cohorts |
| Per health state (1 week) – Autoantibody-positive | | | | |  |
| pc_ww_low_DAS | Remission | €231 (49) | Gamma | Shape= 22, scale= 11 | EAC cohort |
| pc_ww_mod_DAS | Low DAS | €331 (65) | Gamma | Shape= 26, scale= 13 | EAC cohort |
| pc_ww_high_DAS | Moderate/ high DAS | €368 (58) | Gamma | Shape= 40, scale= 9 | EAC cohort |
| pc_ww_no_IA | No IA | €232 (17) | Gamma | Shape= 186, scale= 1 | CSA cohorts |
| Per health state (1 week) – Autoantibody-negative | | | | |  |
| pc_ww_low_DAS | Remission | €144 (53) | Gamma | Shape= 7, scale= 19 | EAC cohort |
| pc_ww_mod_DAS | Low DAS | €364 (74) | Gamma | Shape=24, scale=15 | EAC cohort |
| pc_ww_high_DAS | Moderate/ high DAS | €433 (50) | Gamma | Shape=75, scale=6 | EAC cohort |
| pc_ww_no_IA | No IA | €232 (17) | Gamma | Shape= 186, scale= 1 | CSA cohorts |
| Treatment effect for CSA patients | | | | |  |
| pc_treatment_effect_ lower | Treated autoantibody-positive CSA patients | 75.4% | Triangular | Min= 8%, max=118%, mean = 75.4%) | TREAT EARLIER trial |
| pc_treatment_effect_ lower | Treated autoantibody-negative CSA patients | 94.5% | Triangular | Min=54%, max=126%, mean = 94.5%) | TREAT EARLIER trial |
| Informal care costs |  |  |  |  |  |
| CSA phase |  |  |  |  |  |
| ic_ww_CSA_IA | 5 months in the CSA phase | €201 (20) | Gamma | Shape= 100, scale=2 | CSA cohorts |
| ic_MRI_CSA_IA* | 6.7 months in the CSA phase (relative treatment effect) | €388 (38) | Gamma | Shape=100, scale=4 | CSA cohorts |
| ic_MRI_CSA_IA* | 8 months in the CSA phase (absolute treatment effect) | €438 (44) | Gamma | Shape= 100, scale=4 | CSA cohorts |
| Per health state (3 months) – Autoantibody-positive | | | | |  |
| ic_ww_low_DAS | Remission | €28 (20) | Gamma | shape= 2, scale=15 | EAC cohort |
| ic_ww_mod_DAS | Low DAS | €26 (18) | Gamma | shape= 2, scale=13 | EAC cohort |
| ic_ww_high_DAS | Moderate/ high DAS | €215 (136) | Gamma | shape= 2, scale=86 | EAC cohort |
| ic_ww_no_IA | No IA | €123 (56) | Gamma | shape= 5, scale=26 | CSA cohorts |
| Per health state (3 months) – Autoantibody-negative | | | | |  |
| ic_ww_low_DAS | Remission | €21(17) | Gamma | Shape= 1, scale=14 | EAC cohort |
| ic_ww_mod_DAS | Low DAS | €25 (23) | Gamma | Shape= 1, scale=21 | EAC cohort |
| ic_ww_high_DAS | Moderate/ high DAS | €364 (154) | Gamma | Shape= 6, scale=65 | EAC cohort |
| ic_ww_no_IA | No IA | €123 (56) | Gamma | shape= 5, scale=26 | CSA cohorts |

**This is the same for modified Dixon MRI (hc_MD_MRI_CSA_IA, pc_MD_MRI_CSA_IA, and ic_MD_MRI_CSA_IA), and ultrasound (hc_US_CSA_IA, pc_US_CSA_IA, and ic_US_CSA_IA)).*

**Supplemental file 4. TECHVER-protocol**

The relevant tests were performed to check whether the expected result of the test was met. All tests related to pre-analysis calculations were not performed, because no drug acquisition costs were used to calculate drug prices, the probability of an event was not derived from an odds ratio, relative risk, or hazard ratio, and no survival model and extrapolation were applied.

Tests related to the event-state calculations of the lifetime horizon were not applicable, because a two-year time horizon was used. Also, tests specific for discrete event simulation were not applicable, because we used a decision tree and a Markov model. We only provided our results in quality-adjusted life years and not in life years. The intervention did not impact the life expectancy but only the quality of life, so all tests related to life years were not applicable. The tests related to inflation were not applicable, because we did not correct our inputs for inflation since all costs were based on 2022 estimates. Undiscounted results were not presented in the manuscript, but the tests were performed to check whether the outcomes were as expected. No tunnel states were used, so tests related to tunnel states were not applicable. No time conversion for probabilities was needed, because the data reflected the used cycle length. No disentangled costs were presented, so these tests were not applicable. No adverse events were taken into account separately, so all tests related to AEs were not applicable.

Table S8 shows all the remaining tests. The test was checked when the expected result of the test was matched.

**Table S8. TECHVER-protocol**

| Test description (Please document how the test is conducted, as well) | Expected result of the test | Check |
| --- | --- | --- |
| *Event-state calculations* |  |  |
| Calculate the sum of the number of patients at each health state | Should add up to the cohort size | ☒ |
| Check if all probabilities and number of patients in a state are greater than or equal to zero | Yes | ☒ |
| Check if all probabilities are smaller than or equal to one | Yes | ☒ |
| Compare the number of dead (or any absorbing state) patients in a period with the number of dead (or any absorbing state) patients in the previous periods? | Should be larger | ☒ |
| Set all utilities to zero | No utilities will be accumulated in the model | ☒ |
| Decrease all state utilities simultaneously (but keep event based utility decrements constant) | Lower utilities will be accumulated each time | ☒ |
| Set all costs to zero | No costs will be accumulated in the model at any time | ☒ |
| Put mortality rates to 0 | Patients never die | ☒ |
| Put mortality rate extremely high | Patients die in the first few cycles | ☒ |
| Set the effectiveness, utility and safety related model inputs for all treatment options equal | Same life years and QALYs should be accumulated for all treatment at any time | ☒ Equal diagnostic accuracy leads to same accumulated QALYs |
| In addition to the inputs above, set cost related model inputs for all treatment options equal | Same costs, life years and QALYs should be accumulated for all treatment at any time | ☒ Equal cost when all cost related model inputs are set equal |
| Change around the effectiveness, utility and safety related model inputs between two treatment options | Accumulated life years and QALYs in the model at any time should be also reversed | ☒ |
| Check if the number of alive patients estimate at any cycle is in line with general population life table statistics | At any given age, the % alive should be lower or equal in comparison to the general population estimate | ☒Equal to the general population because only impacted by background mortality |
| Check if the QALY estimate at any cycle is in line with general population utility estimates | At any given age, the utility assigned in the model should be lower or equal in comparison to the general population estimate | ☒ The QALY estimate is lower compared to the general population |
| Calculate the sum of all ingoing and outgoing transition probabilities | Both should be one | ☒ |
| *Decision tree specific:* calculate the sum of the expected probabilities of the terminal nodes | Should sum up to one | ☒ |
| *Result calculations* |  |  |
| Check the incremental life years and QALYs gained results. Are they in line with the comparative clinical effectiveness evidence of the treatments involved? | If a treatment is more effective, it generally results in positive incremental LYs and QALYs in comparison with the less effective treatments | ☒ |
| Check the incremental cost results. Are they in line with the treatment costs? | If a treatment is more expensive, and if it does not have much effect on other costs, it generally results in positive incremental costs. | ☒ |
| Undiscounted results > discounted results | Yes | ☒ |
| Subgroup analysis results: How do the outcomes change if the characteristics of the baseline change? | Better outcomes for better baseline health conditions and worse outcomes for worse health conditions are expected. | ☒ Subgroup analysis ACPA/RF+ vs ACPA/RF-, no such expectations but outcomes seem reliable |
| Could you generate all the results in the report from the model (including the uncertainty analysis results)? | Yes | ☒ |
| Is the reporting and contextualization of the incremental results correct? | The use of the terms such as: “dominant”/ “dominated”/ “extendedly dominated”/ “cost-effective” etc. should be in line with the results. | ☒ |
|  | In the incremental analysis table involving multiple treatments, ICERs should be calculated against the next non-dominated treatment. | ☒ |
| Are the reported ICERs in the fully incremental analysis non-decreasing? | Yes | ☒ |
| Check the discounted value of costs/QALYs after 2 years | Discounted value=undiscounted/(1+r)^2^ | ☒ Did not hold because some costs were not discounted (costs in the decision tree and intervention related costs) |
| Set discount rates to zero | The discounted and undiscounted results should be the same | ☒ |
| Set mortality rate to zero | The undiscounted total life years per patient should be equal to the length of the time horizon | ☒ |
| Set discount rates to a higher value | Total discounted results should decrease | ☒ |
| Set discount rates of costs/effects to an extremely high value | Total discounted results should be more or less the same as the discounted results accrued in the first cycles | ☒ |
| Double the difference in efficacy and safety between new intervention and comparator and report the incremental results. | Approximately twice of the incremental effect results of the base case. If this is not the case : report and explain the underlying reason/ mechanism | Multiple underlying mechanism: delay of event, starting health states and treatment effects which are all dependent on the accuracy of the imaging modality. |
| Do the same for a scenario in which the difference in efficacy and safety is halved. | Approximately halve of the incremental effect results of the base case. If this is not the case : report and explain the underlying reason/ mechanism | Multiple underlying mechanism: delay of event, starting health states and treatment effects which are all dependent on the accuracy of the imaging modality. |
| *Uncertainty analysis calculations* |  |  |
| Are all parameters subject to uncertainty included in the one-way sensitivity analysis (OWSA)? | Yes | ☒ |
| Check if the OWSA includes any parameters associated with joint uncertainty (e.g. parts of a utility regression equation, survival curves with multiple parameters). | No | ☒ |
| Are the upper and lower bounds used in the one-way sensitivity analysis used confidence intervals based on the statistical distribution assumed for that parameter? | Yes | ☒ |
| Are the resulting ICER, incremental costs/QALYs with upper and lower bound of a parameter plausible and in line with a priori expectations? | Yes | ☒ |
|  |  |  |
| Check that all parameters used in the sensitivity analysis have an appropriate associated distributions | Yes | ☒ |
| - upper and lower bounds should surround the deterministic value (i.e. Upper bound ≥ mean ≥ Lower bound) |  | ☒ |
| - standard error and not standard deviation used in sampling |  | ☒ |
| - Lognormal / gamma distribution for hazard ratios and costs/ resource use |  | ☒ |
| - Beta for utilities and proportions/probabilities |  | ☒ |
| - Dirichlet for multinomial |  | ☒ |
| Check PSA output mean costs, QALYs and ICER compared to the deterministic results. Is there a large discrepancy? | No (in general) | ☒ |
| Is(are) the CEAC line(s) in line with the CE scatter plots and the efficient frontier? | Yes | ☒ |
| Does the PSA cloud demonstrate an unexpected behavior or has an unusual shape? | No | ☒ |
| Is the sum of all CEAC lines equal to 1 for all WTP values? | Yes | ☒ |
| Are the explored scenario analyses provide a balanced view on the structural uncertainty? (i.e. not always looking at more optimistic scenarios) | Yes | ☒ |
| Are the scenario analysis results plausible and in line with a priori expectations? | Yes | ☒ |
| Check the correlation between 2 PSA results (i.e. costs/QALYs under the SoC and costs/QALYs under the comparator) | Should be very low (very high) if different (same) random streams are used for different arms | ☒ very high, same random streams used for different arms |
| If a certain seed is used for random number generation (or previously generated random numbers are used), check if they are they scattered evenly between 0-1 when they are plotted? | Yes | ☒Relatively evenly distributed |
| Compare the mean of the parameter samples generated by the model against the point estimate for that parameter, use graphical methods to examine distributions, functions | The sample means and the point estimates will overlap, the graphs will be similar to the corresponding distribution functions (e.g. Normal, Gamma, etc.) | ☒ Yes, checked for a few different parameters |
| Check if sensitivity analyses include any parameters associated with methodological/ structural uncertainty (e.g. annual discount rates, time horizon). | No | ☒ |
| Value of information analysis if applicable: Was this implemented correctly? | Yes | ☒ |
| Which types of analysis? Were aggregated parameters used? Which parameters are grouped together? Does it match the write-up’s suggestions? |  | ☒  Only EVPI was performed |
| Did the electronic model pass the black-box tests of the previous verification stages in all PSA iterations and in all scenario analysis settings? (additional macro can be embedded to PSA code, which stops the PSA when an error such as negative transition probability, is detected) | Yes | ☒ |

**OWSA=one-way sensitivity analysis; ICER = incremental cost-effectiveness ratio; PSA = probabilistic sensitivity analysis; WTP = willingness to pay; CE = cost-effectiveness; CEAC = cost-effectiveness acceptability curve; LY = life years; QALYs = Quality adjusted life years; OR = odds ratio; RR= relative risk; HR = hazard ratio*

**Supplemental file 5. Cost-effectiveness results from the healthcare perspective**

Table S9 provides an overview of the costs and effects, (incremental) NMB, , and ICER based on a full hierarchical analyses, for both autoantibody-positive and -negative patients from the healthcare perspective. For both subgroups, the modified Dixon MRI had the highest NMB and dominates the other strategies. However, in an analysis in which all diagnostic strategies were compared to watchful waiting (Table 2), they were all found to have a positive incremental NMB.

Table S9. Total costs and QALYs, NMB, incremental NMB compared to watchful waiting, and the ICERs: healthcare perspective

|  | Full hierarchical analysis | | | Compared to watchful waiting | | |
| --- | --- | --- | --- | --- | --- | --- |
| Strategy | **Costs (95% CI)** | **QALYs (95% CI)** | **NMB** |  | **iNMB compared to ww** | **ICER compared to ww** |
| Autoantibody-positive | | | | | | |
| Modified Dixon MRI | €5,217 (3,922-7,093) | 1.439 (1.35-1.53) | €66,729 |  | €707 | Dominant |
| Watchful waiting | €5,225 (3,962-7,186) | 1.425 (1.36-1.48) | €66,022 |  |  |  |
| Ultrasound | €5,225 (4,216-6,599) | 1.432 (1.37-1.50) | €66,392 |  | €370 | €63 |
| Conventional MRI | €5,634 (4,347-7,517) | 1.439 (1.35-1.53) | €66,303 |  | €281 | €29,670 |
| Autoantibody-negative | | | | | | |
| Modified Dixon MRI | €4,139 (3,173-5,347) | 1.452 (1.40-1.50) | €68,484 |  | €437 | Dominant |
| Ultrasound | €4,210 (3,284-5,738) | 1.450 (1.40-1.50) | €68,305 |  | €258 | Dominant |
| Watchful waiting | €4,566 (3,287-5,408) | 1.446 (1.40-1.49) | €68,047 |  |  |  |
| Conventional MRI | €4,260 (3,620-5,738) | 1.452 (1.40-1.50) | €68,052 |  | €4 | €49,313 |

**CI=confidence interval, QALYs= quality adjusted life years, NMB=net monetary benefit, iNMB= incremental net monetary benefit, ww= watchful waiting, ICER=incremental cost-effectiveness ratio, and MRI= magnetic resonance imaging.*

Figure S2 showed that, for autoantibody-positive patients, the modified Dixon strategy was preferred. This strategy was cost-effective with a probability of 50% at a WTP threshold of €50,000/QALY. For autoantibody-negative patients, also the modified Dixon MRI strategy was cost-effective with a probability of 62% at a WTP threshold of €50,000/QALY.

**Figure S2. Cost-effectiveness acceptability curves: all strategies compared healthcare perspective**

A. **Autoantibody-positive patients	B. Autoantibody-negative patients**

Figure S3 illustrates the CE-planes and CEACs for each intervention strategy compared to watchful waiting. Among autoantibody-positive patients, all intervention strategies were cost-effective, with a probability of 55% (MRI vs watchful waiting) to 60% (modified Dixon MRI vs watchful waiting) at a WTP threshold of €50,000/QALY. For autoantibody-negative patients, all strategies were costs-effective with probabilities ranging from 50% (MRI vs watchful waiting) to 71% (modified Dixon MRI vs watchful waiting) at a WTP threshold of €50,000/QALY.

**Figure S3. Cost-effectiveness acceptability curves and cost-effectiveness planes: conventional MRI vs watchful waiting.**

A. **CE-plane: Autoantibody-positive patients C. CEAC: Autoantibody-positive patients**

B. **CE-plane: Autoantibody-negative patients**	**D. CEAC: Autoantibody-negative patients**

**CEAC=Cost-effectiveness acceptability curve, and CE-plane= Cost-effectiveness-plane*

**Supplemental file 6: OWSA autoantibody-positive patients**

Outcomes of the OWSA for autoantibody-positive patients. The 10 most influential input parameters.

**Table S10. Outcomes of the OWSA: Autoantibody-positive patients**

| Parameter | Low value | High value | NMB low value | NMB high value |
| --- | --- | --- | --- | --- |
| *Treatment effect productivity costs* | 0.24 | 1.12 | €7,314 | -€699 |
| *Treatment effect quality of life* | -0.08 | 0.12 | -€40 | €5,404 |
| *Quality of life in CSA phase of treated patients* | 0.33 | 0.61 | €456 | €5,467 |
| *Quality of life in CSA phase of untreated patients* | 0.23 | 0.34 | €4,249 | €1,721 |
| *False positives modified Dixon MRI* | 0.10 | 0.58 | €1,408 | €3,823 |
| *True negatives modified Dixon MRI* | 0.42 | 0.90 | €3,823 | €1,408 |
| *Productivity costs in CSA phase of treated patients* | €11,631 | €17,426 | €3,231 | €1,081 |
| *Treatment effect healthcare costs* | 0.47 | 1.35 | €2,650 | €1,423 |
| *Productivity costs in CSA phase of untreated patients* | €7,668 | €11,500 | €2,381 | €3,453 |
| *Informal care costs in CSA phase of treated patients* | €362 | €530 | €3,262 | €2,233 |

**pc=productivity costs, u=utility, CSA=clinically suspect arthralgia, IA= inflammatory arthritis, MRI= magnetic resonance imaging, MD= modified Dixon, ww= watchful waiting, and DAS=disease activity score.*

**Supplemental file 7: OWSA autoantibody-negative patients**

Outcomes of the OWSA for autoantibody-negative patients. The 10 most influential input parameters.

**Table S11. Outcomes of the OWSA: Autoantibody-negative patients**

| Parameter | | Low value | | High value | NMB low value | NMB high value |
| --- | --- | --- | --- | --- | --- | --- |
| *Treatment effect productivity costs* | | 0.64 | | 1.20 | €2,993 | -€1,092 |
| *Treatment effect quality of life* | | -0.05 | | 0.08 | -€386 | €1,769 |
| *True negatives modified Dixon MRI* | | 0.39 | | 0.92 | €1,583 | €127 |
| *Treatment effect initial health state* | | 0.04 | | 0.42 | €450 | €964 |
| *Treatment effect healthcare costs* | | 0.62 | | 1.04 | €797 | €323 |
| *Quality of life in CSA phase of treated patients* | | 0.36 | | 0.60 | €480 | €953 |
| *Proportion initial health state moderate/high DAS* | | 0.63 | | 0.70 | €903 | €448 |
| *Proportion initial health state low DAS* |  | | 0.21 | 0.27 | €426 | €846 |
| *Probability to move from the low DAS health state to the low DAS health state* | | 0.26 | | 0.38 | €960 | €615 |
| *Informal care costs in the remission health state* | | €2 | | €62 | €826 | €502 |

**pc=productivity costs, u=utility, CSA=clinically suspect arthralgia, IA= inflammatory arthritis, MRI= magnetic resonance imaging, MD= modified Dixon, ww= watchful waiting, and DAS=disease activity score.*

**Supplemental file 8: Expected loss curves: Healthcare perspective**

**Figure S4. Expected loss curves: healthcare perspective**

A. ELC: autoantibody-positive patients	B. ELC: autoantibody-negative patients

**CEAC=cost effectiveness acceptability curve, Pr=probability, ELC= expected loss curve, MRI= magnetic resonance imaging, EVPI= expected value of perfect information, and QALY= quality adjusted life year.*

**Supplemental file 9: Scenario analyses**

The results of the probabilistic sensitivity analysis for the three scenario analyses.

**Table S12. Base-case compared to three scenarios: autoantibody-positive patients**

|  | Full hierarchical analysis | | | | Compared to watchful waiting | | |
| --- | --- | --- | --- | --- | --- | --- | --- |
| Strategy | **Costs (95% CI)** | | **QALYs (95% CI)** | **NMB** |  | **iNMB compared to ww** | **ICER compared to ww** |
|  | | **Scenario 1: Utility decrement treated CSA patients** | | | | | |
| Modified Dixon MRI | €32,735 (27,142-38,009) | | 1.427 (1.34-1.52) | €38,634 |  | €2,031 | Dominant |
| Conventional MRI | €33,096 (27,485-38,535) | | 1.427 (1.34-1.52) | €38,266 |  | €1,664 | Dominant |
| Ultrasound | €33,342 (29,592-37,347) | | 1.425 (1.36-1.49) | €37,910 |  | €1,308 | Dominant |
| Watchful waiting | €34,558 (30,990-38,471) | | 1.423 (1.36-1.48) | €36,602 |  | - | - |
|  | | **Scenario 2: Relative delay time-to-IA-diagnosis** | | | | | |
| Modified Dixon MRI | €32,740 (27.392-37,929) | | 1.435 (1.35-1.53) | €38,992 |  | €2,391 | Dominant |
| Conventional MRI | €33,102 (27,742-38,243) | | 1.435 (1.35-1.52) | €38,664 |  | €2,063 | Dominant |
| Ultrasound | €33,470 (29,460-37,941) | | 1.430 (1.36-1.49) | €38,031 |  | €1,430 | Dominant |
| Watchful waiting | €34,570 (31,000-38,484) | | 1.423 (1.36-1.48) | €36,601 |  | - | - |
|  | | **Scenario 3: no additional treatment effect** | | | | | |
| Watchful waiting | €34,473 (30,998-38,341) | | 1.424 (1.36-1.49) | €36,732 |  | - | - |
| Ultrasound | €34,708 (31,995-37,597) | | 1.425 (1.36-1.48) | €36,530 |  | -€202 | €362,957 |
| Modified Dixon MRI | €35,057 (31,748-38,607) | | 1.425 (1.36-1.50) | €36,213 |  | -€520 | €454,720 |
| Conventional MRI | €35,481 (32,183-39,157) | | 1.425 (1.36-1.50) | €35,783 |  | -€949 | €857,523 |
| Scenario 4: Only a treatment effect for individuals developing IA | | | | | | | |
| Modified Dixon MRI | €33,713 (29,338-38,299) | | 1.435 (1.35-1.51) | €38,036 |  | €1,319 | Dominant |
| Ultrasound | €34,030 (30,772-37,226) | | 1.429 (1.37-1.49) | €37,431 |  | €714 | Dominant |
| Conventional MRI | €34,121 (29,820-38,299) | | 1.435 (1.35-1.51) | €37,647 |  | €930 | Dominant |
| Watchful waiting | €34,456 (30,882-38,432) | | 1.423 (1.36-1.48) | €36,717 |  | - | - |

**CI=confidence interval, QALYs= quality adjusted life years, NMB=net monetary benefit, iNMB= incremental net monetary benefit, ww= watchful waiting, ICER=incremental cost-effectiveness ratio, and MRI= magnetic resonance imaging.*

**Figure S5. Cost-effectiveness acceptability curves of all scenarios: autoantibody-positive patients**

a. **Scenario 1: Treatment for all strategy and a utility decrement false positive**

b. **Scenario 2: Relative delay time-to-IA-diagnosis**

c. **Scenario 3: no additional treatment effect**

d. Scenario 4: only treatment effect for individuals developing IA

**Pr=probability, CEAC= cost-effectiveness acceptability curve, MRI= magnetic resonance imaging, and QALY= Quality adjusted life year.*

**Table S13. Base-case compared to three scenarios: autoantibody-negative patients**

|  | Full hierarchical analysis | | | |  | Compared to watchful waiting | | |
| --- | --- | --- | --- | --- | --- | --- | --- | --- |
| Strategy | **Costs (95% CI)** | | **QALYs (95% CI)** | **NMB** |  | **iNMB compared to ww** | **ICER compared to ww** | |
|  | | **Scenario 1: Utility decrement treated CSA patients** | | | | | |  |
| Modified Dixon MRI | €29,156 (25,056-33,328) | | 1.446 (1.40-1.49) | €43,135 |  | €481 | Dominant | |
| Ultrasound | €29, 311(25,529-33,212) | | 1.446 (1.40-1.49) | €42,978 |  | €322 | Dominant | |
| Conventional MRI | €29,595 (25,711-33,714) | | 1.446 (1.40-1.49) | €42,700 |  | €44 | Dominant | |
| Watchful waiting | €29,609 (26,113-33,245) | | 1.445 (1.40-1.49) | €42,656 |  | - | - | |
|  | | **Scenario 2: Relative delay time-to-IA-diagnosis** | | | | | |  |
| Modified Dixon MRI | €29,123 (25,020-33,271) | | 1.451 (1.40-1.50) | €43,451 |  | €796 | Dominant | |
| Ultrasound | €29,298 (25,519-33,192) | | 1.450 (1.41-1.50) | €43,184 |  | €529 | Dominant | |
| Conventional MRI | €29,565 (25,674-33,722) | | 1.452 (1.41-1.49) | €43,019 |  | €364 | Dominant | |
| Watchful waiting | €29,610 (26,115-33.247) | | 1.445 (1.40-1.48) | €42,655 |  | - | - | |
|  | | **Scenario 3: no additional treatment effect** | | | | | |  |
| Watchful waiting | €29,544 (26,270-33,255) | | 1.446 (1.40-1.49) | €42,734 |  | - | - | |
| Ultrasound | €29,710 (26,451-33,451) | | 1.446 (1.40-1.49) | €42,568 |  | -€166 | Dominated | |
| Modified Dixon MRI | €29,772 (26,516-33,396) | | 1.446 (1.40-1.49) | €42,503 |  | -€231 | Dominated | |
| Conventional MRI | €30,195 (26,917-33,887) | | 1.446 (1.40-1.49) | €42,081 |  | -€652 | Dominated | |
| Scenario 4: Only a treatment effect for individuals developing IA | | | | | | | | |
| Watchful waiting | €29,560 (26,263-32,991) | | 1.445 (1.40-1.49) | €42,420 |  | - | - | |
| Ultrasound | €29,688 (26,470-33,105) | | 1.445 (1.40-1.49) | €42,254 |  | -€101 | Dominated | |
| Modified Dixon MRI | €29,688 (26,478-33,181) | | 1.446 (1.40-1.49) | €42,189 |  | -€110 | Dominated | |
| Conventional MRI | €30,137 (26,886-33,546) | | 1.446 (1.40-1.49) | €41,768 |  | -€528 | Dominated | |

**CI=confidence interval, QALYs= quality adjusted life years, NMB=net monetary benefit, iNMB= incremental net monetary benefit, ww= watchful waiting, ICER=incremental cost-effectiveness ratio, and MRI= magnetic resonance imaging.*

**Figure S6. Cost-effectiveness acceptability curves and expected loss curves of all scenarios: autoantibody-negative patients**

a. **Scenario 1: Treatment for all strategy and a utility decrement false positive**

b. **Scenario 2: Relative delay time-to-IA-diagnosis**

c. **Scenario 3: no additional treatment effect**

d. **Scenario** 4**:** Only a **treatment effect** for individuals developing IA

**Pr=probability, CEAC= cost-effectiveness acceptability curve, MRI= magnetic resonance imaging, and QALY= Quality adjusted life year.*
